# Deubiquitinase Mysm1 regulates neural stem cell proliferation and differentiation by controlling Id4 expression

**DOI:** 10.1038/s41419-024-06530-y

**Published:** 2024-02-12

**Authors:** Zhenhua Xu, Qiaozhen Qin, Yan Wang, Heyang Zhang, Shuirong Liu, Xiaotong Li, Yue Chen, Yuqing Wang, Huaqiang Ruan, Wenyan He, Tao Zhang, Xinlong Yan, Changyong Wang, Donggang Xu, Xiaoxia Jiang

**Affiliations:** 1 Beijing Institute of Basic Medical Sciences, 27 Taiping Road, Haidian District, Beijing, 100850 China; 2 Faculty of Environmental and Life Sciences, Beijing University of Technology, Beijing, 100124 China; 3 Anhui Medical University, Hefei, 230032 Anhui China; 4 China National Clinical Research Center for Neurological Diseases, Jing-Jin Center for Neuroinflammation, Beijing Tiantan Hospital, Capital Medical University, Beijing, 100050 China

**Keywords:** Epigenetics and behaviour, Cell death in the nervous system

## Abstract

Neural stem cells (NSCs) are critical for brain development and maintenance of neurogenesis. However, the molecular mechanisms that regulate NSC proliferation and differentiation remain unclear. Mysm1 is a deubiquitinase and is essential for the self-renewal and differentiation of several stem cells. It is unknown whether Mysm1 plays an important role in NSCs. Here, we found that Mysm1 was expressed in NSCs and its expression was increased with age in mice. Mice with Mysm1 knockdown by crossing Mysm1 floxed mice with Nestin-Cre mice exhibited abnormal brain development with microcephaly. Mysm1 deletion promoted NSC proliferation and apoptosis, resulting in depletion of the stem cell pool. In addition, Mysm1-deficient NSCs skewed toward neurogenesis instead of astrogliogenesis. Mechanistic investigations with RNA sequencing and genome-wide CUT&Tag analysis revealed that Mysm1 epigenetically regulated Id4 transcription by regulating histone modification at the promoter region. After rescuing the expression of Id4, the hyperproliferation and imbalance differentiation of Mysm1-deficient NSCs was reversed. Additionally, knockdown Mysm1 in aged mice could promote NSC proliferation. Collectively, the present study identified a new factor Mysm1 which is essential for NSC homeostasis and Mysm1-Id4 axis may be an ideal target for proper NSC proliferation and differentiation.

## Introduction

Neural stem cells (NSCs) are essential for brain development and for proper maintenance of adult neurogenesis. Under precise regulation, NSCs undergo self-renewal or differentiate into diverse types of neural cells and glial cells to ensure proper development of the nervous system and maintain homeostasis in the adult brain [1]. Not surprisingly, NSC dysfunction drives many diseases, including microcephaly, neurological disorders, and age-related disorders [2–6]. Although multiple internal signaling pathways and external environmental factors influencing NSC cell fate have been investigated, the transcriptional regulation of neurogenesis remains unclear.

Histone modification, which affects gene transcription, has been reported to play important roles in NSC homeostasis [7–10]. Myb-like, SWIRM, and MPN domain-containing protein 1 (Mysm1) is a transcriptional regulator that mediates histone deubiquitination [11]. Aberrant expression of Mysm1 leads to different diseases, including bone marrow failure disorder, autoimmune diseases, cancer, and depression [12–18]. By regulating the transcription of some important transcription factors, such as Ebf1 and Id2, Mysm1 controls hematopoietic stem cell (HSC) and common lymphoid progenitors differentiation toward different hematopoietic cells and lymph cells, respectively [12, 19]. Previous studies have also shown that Mysm1 regulates the self-renewal and differentiation of HSCs by regulating Gfi1 expression [13]. After Mysm1 knockout, the dynamic balance of HSCs is destroyed, resulting in depletion of the stem cell pool. Recently, Zhao et al. revealed that Mysm1 overexpression renders HSCs less susceptible to ferroptosis [20]. In addition to regulating HSCs, Mysm1 also plays a critical role in mesenchymal stem cell (MSC) maintenance and differentiation, and Mysm1 knockout MSCs show enhanced autonomous differentiation and accelerated adipogenesis [21]. Bahrami et al. reported that two patients with Mysm1 deficiency show neurocognitive developmental delay [22]. Our previous research revealed the important function of Mysm1 in the brain, highlighting astrocytic Mysm1 as a potential risk factor for depression and as a valuable target for drug discovery to treat depression [16]. Considering the important role of Mysm1 in stem cell homeostasis and brain function, we hypothesized that Mysm1 may regulate NSC homeostasis. Whether and how Mysm1 regulates NSC proliferation and differentiation and then affects brain development and function deserves detail investigation.

In the present study, we found that Mysm1 is essential for normal brain development. Mysm1 knockdown in NSCs leads to NSC proliferation and early differentiation into neurons. Overexpression of Id4 could reverse the hyperproliferation and imbalance differentiation of Mysm1-deficient NSCs. Our data revealed a new factor, Mysm1, in controlling NSC homeostasis and suggested that the Mysm1-Id4 axis may be an ideal target for proper NSC proliferation and differentiation.

## Materials and methods

### Animals

All animal experiments were conducted according to the Guide for the Care and Use of Laboratory Animals by the Administrative Panel on Laboratory Animal Care at the Institute of Basic Medical Sciences (Beijing, China). Floxed Mysm1 (Mysm1^fl/fl^) mice (a kind gift from Dr. Si-Yi Chen at the University of Southern California) were crossed with Nestin-Cre (Nes^cre^) transgenic mice (a kind gift from Dr. Zeng-Qiang Yuan at the Beijing Institute of Basic Medical Sciences). All lines were on the C57BL/6 background. Mysm1 conditional knockout (Nes^cre^; Mysm1^fl/fl^) mice were generated by crossing Mysm1^fl/f^ mice with Nestin-Cre mice. Offspring were genotyped by polymerase chain reaction (PCR) of genomic DNA extracted from the mouse tail and analyzed with the primers shown in Supplementary Table 1. PCR was performed using a Mastercycler® gradient thermal cycler (Eppendorf, Hamburg, Germany) with 2xTaq PCR MasterMix (Aidlab Biotechnologies). The thermocycler conditions were 95 °C for 5 min; and 35 cycles at 95 °C for 30 s, 57.5 °C for 30 s, and 72 °C for 30 s; and final extension at 72 °C for 5 min. In all experiments, Mysm1^fl/fl^ littermates served as controls. The knockout efficiency was verified by quantitative real-time polymerase chain reaction (qRT-PCR) analysis and western blot analysis of NSCs prepared from control (CTRL) and conditional knockout (cKO) mice. C57BL/6 mice (14–15 months of age) were used as aged models, and pathological analysis was performed 3 weeks after virus microinjection. All C57BL/6 mice were purchased from Charles River (Beijing, China). Four to five mice were housed in a plastic cage (300 × 120 × 170 mm) and maintained in a circadian cycle of 12 h of light (8:00 a.m.–20:00 p.m.) and 12 h of dark (20:00 p.m.–8:00 a.m.) with an adequate food and water supply.

### Cell cultures

NSCs were prepared from E12.5 cortices of mice according to previous protocols [23]. Isolated NSCs were cultured in complete DMEM-F12 medium (Gibco) supplemented with 1 × B27 supplement (Gibco), 2 mM L-glutamine (Gibco), 20 ng/mL EGF (PeproTech), and 20 ng/mL bFGF (PeproTech) [24]. Cells were maintained as primary neurospheres in uncoated dishes for 5 days. To passage neurospheres, 0.25% trypsin (Sigma) was used to digest neurospheres into single cells, and then single cells were incubated in serum-free medium for another 7 days (passage 1 neurospheres). Experiments were performed with cultured cells of passages 2–3.

### Lentivirus and adeno-associated virus

NSCs were infected with lentivirus and screened by using puromycin to stably overexpress Mysm1 (pcSLenti-EF1-EGFP-P2A-Puro-CMV-Mysm1-3xFLAG- WPRE), overexpress Id4 (pcSLenti-EF1-EGFP-P2A-Puro-CMV-Id4-3xFLAG- WPRE), or knockdown Mysm1 (HBLV-m-Mysm1 shRNA1-ZsGreen-PURO) according to the manufacturer’s instructions. The lentiviruses to overexpress Mysm1 or Id4 were purchased from OBiO Technology Co., Ltd. (Shanghai, China). Mice were infected with adeno-associated virus (AAV) to stably knock down Mysm1 (HBAAV2/9- GFAP-mir30-m-Mysm1-NULL) in NSCs at the injection site according to the manufacturer’s instructions. The lentivirus to knock down Mysm1 and AAV was purchased from Hanbio Co., Inc. (Shanghai, China). The top strand sequence of the shRNA for Mysm1 was GATCCGCCACCAATCAAGGAGAATTATctcgagATA ATTCTCCTTGATTGGTGGTTTTTTG, the bottom strand sequence of the shRNA for Mysm1 was AATTCAAAAAACCACCAATCAAGGAGAATTATctcgagATAA TTCTCCTTGATTGGTGGCG, the transcript sequence for overexpressing Mysm1 was NM_177239.3, and the transcript sequence for overexpressing Id4 was NM_031166.3. Details of virus vectors are shown in Supplementary Fig. 6.

### qRT-PCR

Total RNA from cells and tissues was extracted and isolated by TRIzol (Invitrogen). Total RNA was extracted by chloroform extraction and isopropanol precipitation according to the manufacturer’s recommendations. Reverse transcription was performed using ReverTra Ace qPCR RT Master Mix (Toyobo, 037400). Quantitative PCR was performed with 2×T5 Fast qPCR Mix (SYBR) (TSINGKE, TSE202). Each amplification cycle consisted of an initial step at 95 °C (5 min) followed by 40 cycles of denaturation at 95 °C for 15 s, annealing at 60 °C for 1 min, and extension at 72 °C for 30 s. GAPDH or β-actin was used as an internal control. The primer sequences for qRT-PCR are listed in Supplementary Table 2.

### Western blot analysis

Cells and tissues were homogenized in lysis buffer (RIPA) on ice for 40 min and subsequently centrifuged at 12,000 rpm for 5 min at 4 °C. The supernatants were then transferred to a clean 1.5 mL tube. Samples containing 30 μg of protein were separated using 12% SDS–PAGE gels. Proteins were transferred onto polyvinylidene difluoride membranes in cold buffer (25 mM Tris Base and 192 mM glycine) by electrotransfer for 1.5 h, and the membranes were then incubated in TBS buffer containing 5% milk for 1 h at 22–24 °C. The membrane was probed at 4 °C overnight with the following primary antibodies: Mysm1 (ab193081, Abcam), Mcm2 (ab108935, Abcam), phosphorylated p53 (p-p53, #82530, Cell Signaling Technology), p53 (#9282, Cell Signaling Technology), Puma (55120-1-AP, Proteintech), Bax (#41162, Cell Signaling Technology), Map2 (ab32454, Abcam), doublecortin (Dcx, #91954, Cell Signaling Technology), Aldh1l1 (#85828, Cell Signaling Technology), Gfap (bs-0199R, Bioss), Olig2 (#65915, Cell Signaling Technology), NG2 (ab255811, Abcam), Id4 (sc-365656, Santa Cruz Biotechnology), Bcl-2 (#3498S, Cell Signaling Technology), β-Actin (AC004, ABclonal) and GAPDH (AC001, ABclonal). After three washes, the membranes were incubated with secondary antibodies in TBS buffer for 1 h at 22–24 °C. Enhanced chemiluminescence (APPLYGEN) was used to visualize the immunoreactive bands. The secondary antibodies included HRP goat anti-rabbit IgG (AS029, ABclonal) and HRP goat anti-mouse IgG (AS003, ABclonal).

### Cell cycle analysis

NSCs expressing Mysm1 shRNA (shMysm1) and scrambled shRNA (shCtrl) were harvested, centrifuged, and washed three times with PBS. Cells were then suspended and fixed in 70% ethanol at 4 °C for 24 h. After washing twice with PBS, fixed cells were resuspended in 500 μL of PBS containing 50 μg/mL PI, 100 μg/mL RNase A, and 0.2% Triton X‐100, followed by incubation for 30 min. The cell cycle was analyzed using NovoCyte TM flow cytometry (Agilent, USA).

### CUT&Tag

CUT&Tag was performed with a Hyperactive Universal CUT&Tag Assay Kit for Illumina (TD903, Vazyme Biotech) according to the manufacturer’s recommended protocol [25]. In brief, NSCs stably expressing Mysm1-Flag and induced astrocytes were collected to extract nuclei in NE buffer and then combined with ConA Beads. Subsequently, cells were resuspended in antibody buffer and incubated with primary antibodies against Flag (F1804, Sigma‒Aldrich) and secondary antibodies (Ab206-01, Vazyme Biotech). The samples were incubated with pA/G-Tnp transposase. After transposon activation and tagmentation, DNA was isolated, amplified, and purified to construct a library. The library for sequencing was constructed, and VAHTS DNA Clean Beads (N411, Vazyme Biotech) were used for purification steps. The library was quantified with a VAHTS Library Quantification Kit for Illumina (Vazyme Biotech) and sequenced on an Illumina NovaSeq 150PE. All cells were tested for mycoplasma contamination before experiment.

### CUT&RUN-qPCR

CUT&RUN-qPCR is a new method to extract the DNA bound to protein. NSCs stably expressing Mysm1-Flag and induced astrocytes were harvested and treated according to the manufacturer’s instructions by using a Hyperactive pG-MNase CUT&RUN Assay Kit for PCR/qPCR (HD101, Vazyme Biotech). In brief, cells were collected to combined with ConA Beads Pro. Subsequently, cells were resuspended in antibody buffer and incubated with primary antibodies against Flag (F1804, Sigma‒Aldrich), ubiquityl-Histone H2A (#05-678, Millipore), Tri-Methyl-Histone H3 (Lys4) (#9751, Cell Signaling Technology), or Acetyl-Histone H3 (Lys9) (#9649, Cell Signaling Technology). Antibody-protein complexes were incubated with pG-MNase Enzyme. Then fragmentation was terminated and released by Stop Buffer containing Spike in DNA. The DNAs were extracted by FastPure gDNA Mini Columns and subjected to qPCR. The Spike in DNA was used as a reference and the primer sequences for CUT&RUN-qPCR are listed in Supplementary Table 3. All cells were tested for mycoplasma contamination before experiment.

### Immunofluorescent staining

Mouse brains were dissected and fixed with 4% paraformaldehyde in PBS for 3 days at room temperature (RT). The brains were then dehydrated with a sucrose gradient (10, 20, and 30%) in PBS. The 40-μm coronal brain slices were sectioned with a Leica CM3050S (Leica). For cultured NSCs, cells were seeded onto poly-L-lysine-coated 24-well chamber slides (Corning) and fixed with 4% PFA for 10 min at RT. Brain slices and cultured NSCs were permeabilized in PBS twice and incubated in blocking buffer (PBS containing 0.4% Triton X-100, 2% goat serum, and 1% bovine serum albumin) for 1 h at room temperature. The slices were then incubated with the following primary antibodies: Nestin (MA1-110, Invitrogen), Mysm1 (ab193081, Abcam), Gfap (#MAB360, Millipore), Map2 (ab32454, Abcam), Ki67 (ab15580, Abcam), Tbr2 (ab216870, Abcam), Doublecortin (Dcx, #91954, Cell Signaling Technology), NeuN (ab104225, Abcam), S100β (ab52642, Abcam), Olig2 (#65915, Cell Signaling Technology), NG2 (ab255811, Abcam), Active Caspase-3 (bsm-33199 m, Bioss), β3-tubulin (ab52623, Abcam), tyrosine hydroxylase (TH, ab137869, Abcam), VGLUT2 (ab79157, Abcam). The slices were then incubated with the following appropriate secondary antibodies for 1 h at room temperature: Alexa Fluor® 488 AffiniPure F(ab’)_2_ Fragment Donkey Anti-Mouse IgG (H + L) (715-546-150, Jackson Immunoresearch), Cy™3 AffiniPure Donkey Anti-Rabbit IgG (H + L) (711-165-152, Jackson Immunoresearch), Alexa Fluor @488-AffiniPure Goat Anti-Rabbit IgG (H + L) (SGAR488, YiShan Biotech), or Alexa Fluor @594-AffiniPure Goat Anti-Mouse lgG (H + L) (SGAM594, YiShan Biotech). Images of immunostainings were captured and processed with or without Z stacks on a confocal microscope (Leica). Positive cells were counted and analyzed using ImageJ software.

### Neurosphere assay

A neurosphere assay was performed to test the self-renewal capacity of cultured NSCs. NSCs precultured directly from dissociated tissue in the form of neurospheres were digested into single cells by trypsin and plated at a density of 500 cells per 500 µL of medium in each well of a 24-well plate to form primary neurospheres. The diameters of neurospheres were quantified in the two groups at 1, 3, 5, and 7 days.

### Differentiation assay

NSCs were resuspended in differentiation medium, seeded onto poly-L-lysine-coated 24-well chamber slides as monolayers at a density of 1 × 10^5^/mL for immunostaining analysis, or seeded onto poly-L-lysine-coated six-well plates as monolayers at a density of 1 × 10^5^/mL for western blot analysis. For directed differentiation, NSCs were incubated in the following media: DMEM-F12 medium (Gibco) supplemented with 1 × B27 supplement and 1% FBS for neuronal differentiation; DMEM supplemented with 1 × N-2 supplement (Gibco), 2 mM L-glutamine, and 1% FBS for astrocyte differentiation; and DMEM-F12 medium (Gibco) supplemented with 1 × B27 supplement, 1% FBS, and 30 ng/mL triiodothyronine (T3) solution (supelco) for oligodendrocyte differentiation. The following primary antibodies were used to identify various differentiated cells: Map2, β3-tubulin, and Dcx for neural differentiation; Gfap, S100β, and Aldh1l1 for astrocyte differentiation; Olig2 and NG2 for oligodendrocyte differentiation.

### Stereotaxic surgery and virus injection

Mice (14–15 months old) were anesthetized using 2,2,2-tribromoethanol (Sigma, 240 mg/kg of body weight) and placed on a stereotactic frame (RWD). Virus was stereotactically injected into the subventricular zone bilaterally as shown in Fig. 6E (AP, +0.2 mm from bregma; ML, ±1.2 mm; DV, −2.2 mm from the brain surface) with a pressure microinjector (Hamilton 701RN) at a slow rate of 0.2 μL/min. The injection needle was withdrawn 10 min after the infusion. EdU injections were performed 3 weeks after AAV injections. All animals were intraperitoneally injected with 200 mg/kg EdU. Mice were sacrificed 2 h after EdU injection for furection by a BeyoClick™ EdU-594 cell proliferation detection kit (Beyotime).

### High-throughput RNA sequencing (RNA-Seq)

Total RNA was extracted from cells using TRIzol reagent (Invitrogen, 10296010) according to the manufacturer’s protocol. RNA purity and quantification were evaluated using a NanoDrop 2000 spectrophotometer (Thermo Scientific, USA). RNA integrity was assessed using the Agilent 2100 Bioanalyzer (Agilent Technologies, Santa Clara, CA, USA). Libraries were constructed using the TruSeq Stranded mRNA LT Sample Prep Kit (Illumina, San Diego, CA, USA) according to the manufacturer’s instructions. Transcriptome sequencing and analysis were conducted by Majorbio Co., Ltd. (Shanghai, China). The libraries were sequenced on an Illumina HiSeq X Ten platform, and 150 bp paired-end reads were generated. Raw data (raw reads) in fastq format were first processed using Trimmomatic, and the low-quality reads were removed to obtain clean reads.

### Evaluation of electrophysiological activity

Each multielectrode arrays (MEA) sensor (multichannel systems-MEA2100, Harvard Bioscience Co., Ltd, China) contained an array of 60 electrodes arranged in an 8 × 8 grid (30 μm diameter) spaced 200 μm apart. NSCs were resuspended in neural differentiation medium and implanted in the electrode region previously coated with poly-L-lysine (12 μg/ml) and 20 μl laminin (Sigma Aldrich) at a density of 1 × 10^6^ cells/plate. On Day 7, the spontaneous activity of these cells was recorded using MEA. During recordings, the MEAs were covered with a breathable sealing plate membrane to stabilize osmolarity (BioTss, Co., Inc., China). A temperature controller (multichannel systems-TCO2, Harvard Bioscience Co., Ltd, China) maintained the temperature of the MEA at 37 °C. The system hardware consisted of an amplifier that was interfaced with a PC. MC_Rack software was used to collect spontaneous network activity data. Signals from the amplifier were digitized at a rate of 20 kHz and high-pass filtered (cutoff frequency of 200 Hz). A software-based spike detector was used to detect spontaneous events that exceeded a threshold of 15 μV. Action potentials were distinguished from noise using a voltage threshold five times the standard deviations. The typical peak-to-peak noise level of MEA electrodes was ~5–8 μV. Only electrical signals with physiologically defined features, such as spikes exceeding the threshold of 15 μV with well-defined waveform characteristics, were included in the analysis. The spike count files generated from the recordings were used to calculate the number of spikes/active electrode.

### Average optical density analysis

ImageJ software was used to analyze the average optical density (AOD) (integrated optical density/area) to indicate the level of protein expression. Three different visual immunofluorescence fields were acquired from different tissue sections, and the average AOD was calculated. All tissue sections were stained within one batch with the same imaging threshold and exposure time to ensure maintained consistency for image analysis.

### Cell viability analyses

Analyses of NSC viability were performed by live-dead staining using a Live/Dead stain kit (Thermo Fisher, L3224) after seeding onto poly-L-lysine-coated 24-well chamber slides and treating with 2 µM Apoptosis Activator 2 (M2403, AbMole) for 90 min. Briefly, cells on discs were stained with 2 × 10^−6^ M calcein AM and 10 × 10^−6^ M EthD-1 followed by incubation for 30 min and observation under an Olympus fluorescence microscope. The dead and living cells were stained red and bright green, respectively. The results are expressed as the live/dead cell rate.

### Statistical analysis

The sample sizes required for the experiments were estimated based on the preliminary results. All experiments were performed and analyzed by the same experimenter who was blinded to the animals’ genotype or group treatment under assessment. The studies were blinded during data collection and quantification. The data shown in the figures reflect several independent experiments performed on different days. No data were excluded. No statistical methods were used to predetermine sample size in other experiments. The sample sizes are included in the figure legends and the statistical parameters are listed in the figures and figure legends. All data are presented as the mean with standard error of the mean (mean ± SEM). Statistical analysis was performed using GraphPad Prism version 7 software. Two-tailed unpaired Student’s *t* test was used to compare two conditions. One-way ANOVA was used to compare above three conditions. Significance was set as *p* < 0.05 and expressed as *p < 0.05, **p < 0.01, ***p < 0.001, *and* ****P < 0.0001.

## Results

### Mysm1 is expressed in NSCs and Mysm1-deficient NSCs demonstrate hyperproliferation and preferential neuronal differentiation

**Fig. 1:**
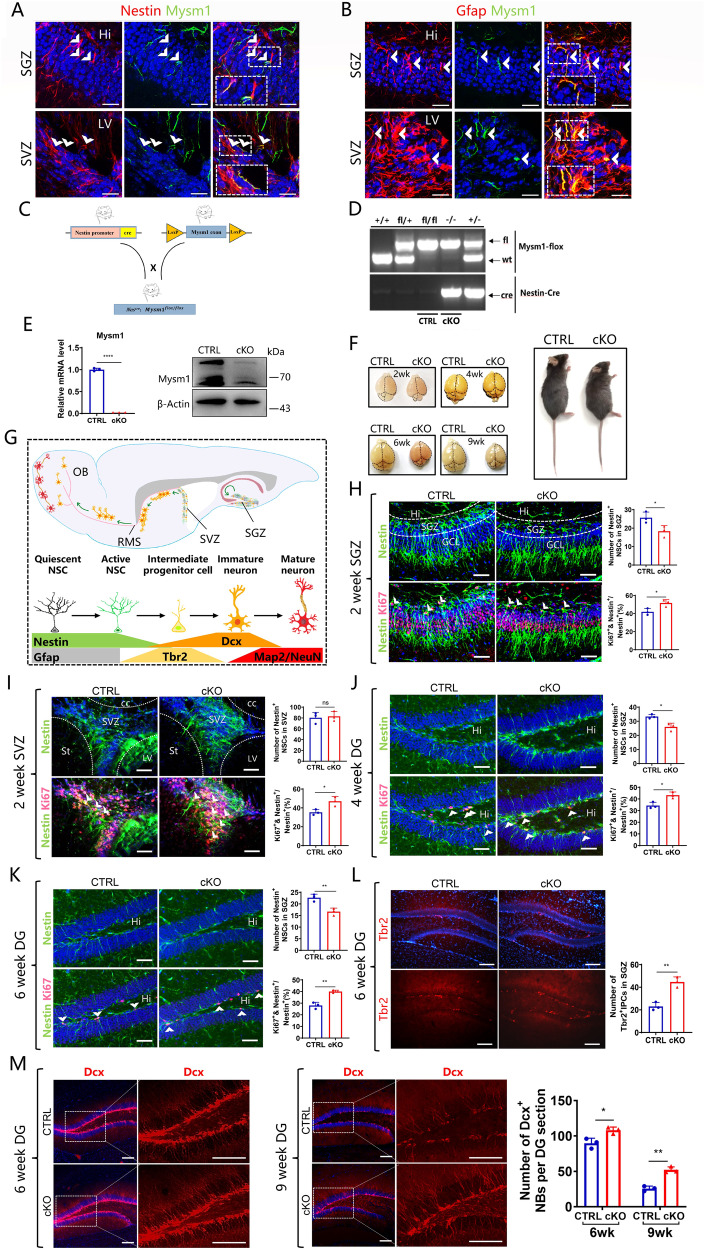
Mysm1 is expressed in NSCs, and Mysm1-deficient mice reveal overactivation and preferential neuronal differentiation of NSCs. **A**, **B** Immunofluorescence for the indicated markers in the SGZ and SVZ of 4-week-old mice. Mysm1 was coexpressed in some Nestin^+^ NSCs or rGfap^+^ NSCs (white arrows). The boxed areas are shown in detail as insets. **C** Nestin-Cre mice were crossed with Mysm1^fl/fl^ mice to generate Mysm1 conditional knockout embryos. **D** Genotyping was performed with the indicated primers. The upper band indicates floxed, and the lower band indicates wild-type. **E** The knockout efficiency was verified by qRT-PCR analysis and western blot analysis of NSCs prepared from CTRL and cKO mice (*n* = 3). **F** Comparisons of the appearance of the brain parenchyma (left) and the external morphology (right, 9-week-old) of CTRL and Mysm1 cKO mice. **G** Schematic diagram of NSC development in neurogenesis niches. New neurons in the OB and DG of the adult brain are generated from NSCs in the SVZ and SGZ, respectively. Immunofluorescence (left) for Nestin, Ki67, and DAPI in the SGZ (**H**) or SVZ (**I**) of 2-week-old CTRL and Mysm1 cKO mice. Number (right upper) of Nestin^+^ NSCs and percentage (right lower) of Ki67^+^ Nestin^+^ NSCs among total Nestin^+^ NSCs in the SGZ (**H**) or SVZ (**I**) of 2-week-old CTRL and Mysm1 cKO mice (*n* = 3). Immunofluorescence (left) for Nestin, Ki67, and DAPI in the SGZ of 4-week-old (**J**) and 6-week-old (**K**) CTRL and Mysm1 cKO mice. Number (right upper) of Nestin^+^ NSCs and percentage (right lower) of Ki67^+^ Nestin^+^ NSCs among total Nestin^+^ NSCs in the SGZ of 4-week-old (**J**) and 6-week-old (**K**) CTRL and Mysm1 cKO mice (*n* = 3). **L** Immunofluorescence (left) for Tbr2 and DAPI in the DG of 6-week-old CTRL and Mysm1 cKO mice. Quantification (right) of Tbr2^+^ cells per DG section in 6-week-old CTRL and Mysm1 cKO mice (*n* = 3). **M** Immunofluorescence (left and middle) for Dcx and DAPI in the DG of CTRL and Mysm1 cKO mice at 6 weeks and 9 weeks. The boxed areas are shown in detail on the right side. Quantification (right) of Dcx^+^ cells per DG section in CTRL and Mysm1 cKO mice at 6 weeks and 9 weeks (*n* = 3). SGZ subgranular zone, SVZ subventricular zone, Hi Hilus, LV lateral ventricles, GCL granule cell layer, DG dentate gyrus, cc corpus callosum, St striatum, wk week. Data are presented as the mean value ± SEM. ns not significant; *p < 0.05, **p < 0.01, and ****P < 0.0001. Scale bars represent 20 μm (**A**, **B**), 50 μm (**H**–**K**), and 100 μm (**L**, **M**).

To investigate the function of Mysm1 in NSCs, immunofluorescence staining for evaluating Mysm1 expression was performed first. NSCs persist in the adult mouse brain in the subventricular zone (SVZ) of the lateral ventricles and the subgranular zone (SGZ) of the hippocampal dentate gyrus (DG) [26]. In the SGZ and SVZ, Mysm1 was coexpressed in some Nestin^+^ NSCs and radial morphology Gfap^+^ (rGfap^+^) NSCs (Fig. 1A, B). To assess the functions of Mysm1 in the mouse brain, we hybridized Nestin-Cre mice with Mysm1 floxed (Mysm1^fl/fl^) mice to obtain Mysm1 cKO (Nes^cre^;Mysm1^fl/fl^) mice (Fig. 1C). Mysm1^fl/fl^ littermates without Nestin-Cre were used as the CTRL mice. Genotyping was performed to identify the cKO group and CTRL group (Fig. 1D), qRT-PCR and western blot analyses showed that the expression of Mysm1 at the mRNA and protein levels was significantly reduced in NSCs prepared from cKO mice compared to NSCs prepared from CTRL mice (Fig. 1E). Compared to CTRL littermates, Mysm1 cKO mice had smaller brains and bodies. The brains from Mysm1 cKO mice were smaller than those from CTRL littermates, suggesting that the right brain development of Mysm1 cKO mice was disturbed (Fig. 1F and Supplementary Fig. 1A).

Developing neurons express distinct markers during their maturation process [27]. Factors expressed at specific stages in the development from NSCs to neurons were evaluated (Fig. 1G). To explore the function of Mysm1 in NSCs, brain sections of CTRL and Mysm1 cKO mice were immunohistochemically analyzed. Compared to CTRL mice, the number of Nestin^+^ NSCs in the SGZ of 2-week-old Mysm1 cKO mice was significantly decreased, but there was no significant change in the number of Nestin^+^ NSCs in the SVZ (Fig. 1H, I). Surprisingly, the proportion of Nestin^+^ Ki67^+^ NSCs in Nestin^+^ NSCs increased significantly in the SGZ and SVZ of Mysm1 cKO mice (Fig. 1H, I). An increased proportion of Nestin^+^ Ki67^+^ NSCs in Nestin^+^ NSCs and a decreased number of Nestin^+^ NSCs were also observed in the SGZ from 4-week-old and 6-week-old Mysm1 cKO mice (Fig. 1J, K). In addition, the number of rGfap^+^ NSCs decreased in the SGZ of 6-week-old and 9-week-old Mysm1 cKO mice (Supplementary Fig. 1B, C). During NSC differentiation, the Nestin and Gfap stem cell markers were decreased, and Tbr2, a marker for intermediate progenitor cells, was increased. We next examined the expression of Tbr2 in the hippocampal DG from 6-week-old CTRL and Mysm1 cKO mice, which demonstrated that the number of Tbr2^+^ cells in the DG of Mysm1 cKO mice was significantly increased (Fig. 1L). Compared to CTRL mice, the number of Dcx^+^ cells in the DG of Mysm1 cKO mice was significantly increased at 6 and 9 weeks (Fig. 1M), and the average optical density of Gfap in the DG of Mysm1 cKO mice decreased at 6 and 9 weeks (Supplementary Fig. 1B and Supplementary Fig. 1C). These results suggested that the NSCs of Mysm1 cKO mice are more likely to differentiate into neuronal lineages. These findings suggested that the early depletion of the NSC pool in Mysm1 cKO mice may cause disordered neurogenesis. Together, these results revealed that the knockdown of Mysm1 may lead to the overactivation of NSCs, which temporarily enhances neurogenesis but depletes the NSC pool, resulting in a neurogenesis disorder.

### Mysm1 knockdown promotes the proliferation and increases susceptibility to apoptosis of NSCs in vitro

**Fig. 2:**
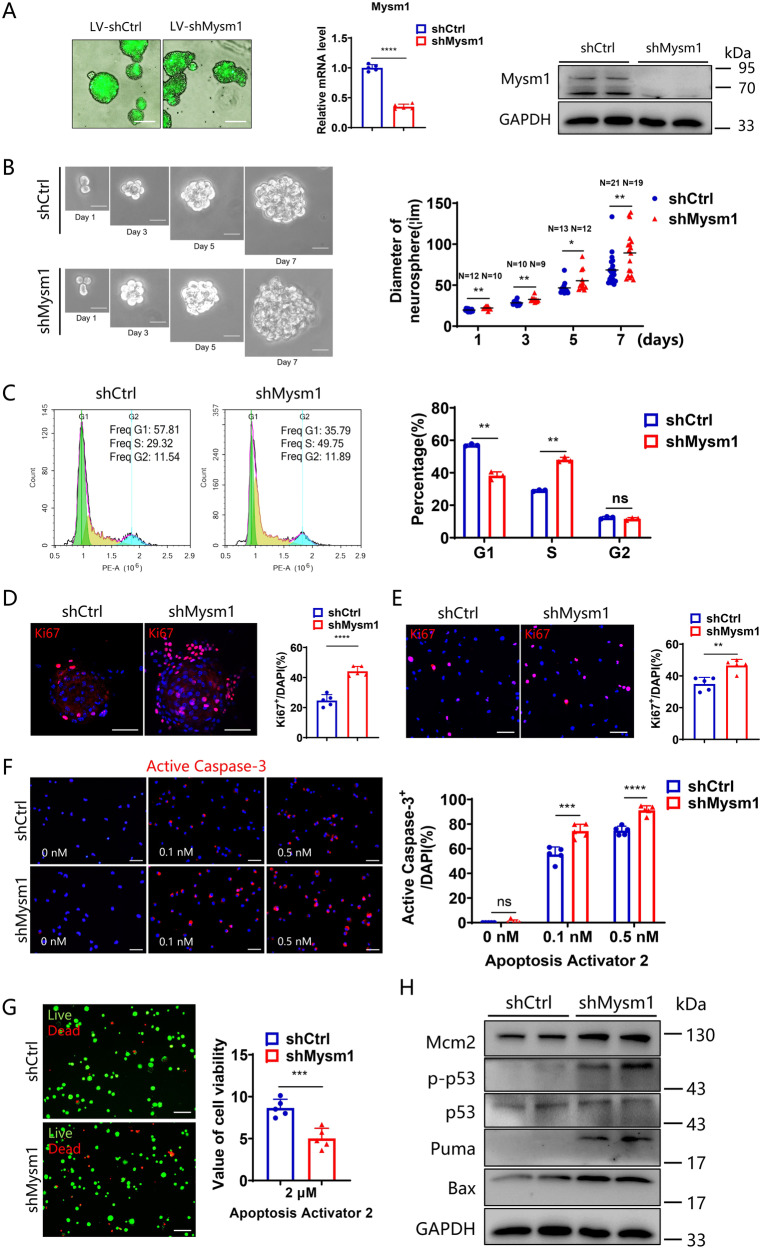
Mysm1 knockdown promotes the proliferation and apoptosis of NSCs in vitro. **A** After infection of WT NSCs with knockdown control and Mysm1 knockdown lentiviruses, the knockdown efficiency was verified by green fluorescence (left), qRT-PCR analysis (middle, *n* = *5*), and western blot analysis (right). **B** (Left) Single shCtrl and shMysm1 NSCs were separated by serial dilution, and neurosphere formation was induced for 7 days in vitro. (Right) The relative size of neurospheres grown to 7 days was quantified by ImageJ software. **C** Flow cytometry analysis using propidium iodide (PI) was used to evaluate the cell cycle of shCtrl and shMysm1 NSCs (*n* = 3). **D** Immunofluorescence (left) for Ki67 and DAPI in neurospheres containing shCtrl and shMysm1 NSCs. Percentage (right) of Ki67^+^ NSCs among total cells per neurosphere containing shCtrl and shMysm1 NSCs (*n* = 5). **E** Immunofluorescence (left) for Ki67 and DAPI in shCtrl and shMysm1 NSCs. Percentage (right) of Ki67^+^ NSCs among total shCtrl and shMysm1 NSCs (*n* = 5). **F** Immunofluorescence (left) for Active Caspase-3 and DAPI in shCtrl and shMysm1 NSCs unstimulated or stimulated by different concentrations of Apoptosis Activator 2. Percentage (right) of Active Caspase-3^+^ cells among total shCtrl and shMysm1 NSCs (*n* = 5). **G** Representative staining images (left) and quantification of cell viability (right) according to calcein-AM (green)/ethidium homodimer (red) staining of shCtrl and shMysm1 NSCs stimulated by 2 μM Apoptosis Activator 2 (*n* = 5). **H** Western blot analysis of the indicated protein in shCtrl and shMysm1 NSCs. Data are presented as the mean value ± SEM. ns not significant; *P < 0.05*,* **P < 0.01*,* ***P < 0.001*,* and ****P < 0.0001. Scale bars represent 50 μm (**A**) and 20 μm (**B**, **D**, **E**, **F**, and **G**).

To further investigate whether Mysm1 regulates the proliferation and differentiation of NSCs, a lentivirus expressing Mysm1 shRNA and green fluorescent protein was generated to knockdown endogenous Mysm1 in cultured NSCs. Green fluorescence was found on almost all the cells, indicating successful transduction. qRT-PCR and western blot analyses showed that the expression of Mysm1 at the RNA and protein levels was significantly reduced in shMysm1 NSCs compared to shCtrl NSCs (Fig. 2A). After successful transduction, shCtrl and shMysm1 NSCs were cultured for 7 days in vitro, and the diameters of neurospheres growing from Day 1 to Day 7 were measured by ImageJ software. The results showed that the diameters of neurospheres from shMysm1 NSCs were larger than those from shCtrl NSCs after 5 days of culture (Supplementary Fig. 2). Next, a single-cell clonal neurosphere formation assay was performed using the second generation of NSCs transduced with lentivirus to assess Mysm1 function in NSC self-renewal activity. The neurospheres of shMysm1 NSCs were significantly larger than those of shCtrl NSCs on Days 1, 3, 5, and 7 of culture (Fig. 2B). In addition, flow cytometry was used to analyze the cell cycle of shCtrl and shMysm1 NSCs. The proportion of shMysm1 NSCs in S phase was significantly higher than that of shCtrl NSCs (Fig. 2C). At the same time, we also evaluated the proportion of Ki67^+^ NSCs in neurospheres and in the single-cell suspension. Similarly, the proportion of Ki67^+^ NSCs in shMysm1 NSCs cells was significantly higher than that in shCtrl NSCs (Fig. 2D, E). In addition to proliferation, NSC apoptosis after Mysm1 knockdown was assessed by Active Caspase-3, a marker commonly used to identify apoptosis. shCtrl and shMysm1 NSCs were stimulated with 0.1 nM and 0.5 nM Apoptosis Activator 2, and the percentage of Active Caspase-3^+^ cells after stimulation for 90 min was calculated. As shown in Fig. 2F, there were no differences between shCtrl and shMysm1 NSCs without stimulation; however, the proportion of Active Caspase-3^+^ cells in shMysm1 NSCs was significantly higher than that in shCtrl NSCs after treatment with Apoptosis Activator 2. To detect cell death, Apoptosis Activator 2 at 2 µM was used to stimulate NSCs after lentiviral transduction. Live and dead assays were performed using calcein-AM (green, live)/ethidium homodimer (red, dead) staining. Increased red staining, indicating increased cell death, was observed in shMysm1 NSCs compared to control NSCs, which indicated that deletion of Mysm1 reduced the viability of NSCs (Fig. 2G). Western blot analysis showed that deletion of Mysm1 significantly increased the expression of Mcm2, phosphorylated p53 (p-p53), Puma, and Bax in NSCs (Fig. 2H). Collectively, these data revealed an important role of Mysm1 in regulating the proliferation and apoptosis of NSCs in vitro.

### Mysm1 knockdown skews NSCs toward neuronal differentiation in vitro

**Fig. 3:**
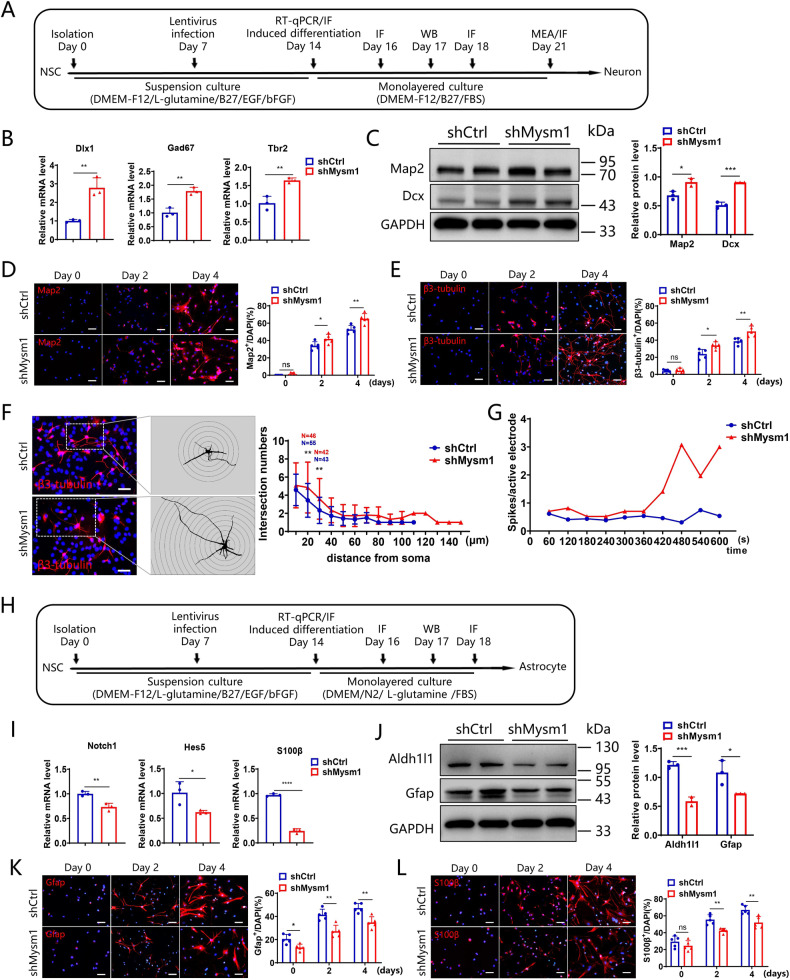
Mysm1 knockdown skews NSCs toward neuronal differentiation in vitro. **A** Time schedule for the in vitro culture of neurospheres and induction of NSCs into neurons, experiments at different time points are marked on the timeline. **B** qRT-PCR analysis of the indicated transcripts in shCtrl and shMysm1 NSCs (*n* = 3). **C** Western blot analysis (left) of the indicated proteins in shCtrl and shMysm1 NSCs after 3 days of differentiation into neurons, and the protein level was normalized to GAPDH (right, *n* = 3). Immunofluorescence (left) for Map2 (**D**), β3-tubulin (**E**), and DAPI in shCtrl and shMysm1 NSCs under undifferentiated and differentiated conditions. Percentage (right) of Map2^+^ (**D**) or β3-tubulin^+^ (**E**) cells among total cells of shCtrl and shMysm1 NSCs (*n* = 5). **F** Immunofluorescence for β3-tubulin and DAPI in shCtrl and shMysm1 NSCs after 7 days of differentiation into neurons. After reconstructing and visualizing the neurites, Sholl analysis was performed to determine the length and number of neurites. **G** A multielectrode array system was used to examine the electrophysiological activity, and the average spikes of each active electrode per minute were calculated. The displayed figure is representative of two experiments. **H** Time schedule for the in vitro culture of neurospheres and NSCs induced to astrocytes, experiments at different time points are marked on the timeline. **I** qRT-PCR analysis of the indicated transcripts in shCtrl and shMysm1 NSCs (*n* = 3). **J** Western blot analysis (left) of the indicated proteins in shCtrl and shMysm1 NSCs after 3 days of differentiation into astrocytes, and the protein level was normalized to GAPDH (right, *n* = 3). Immunofluorescence (left) for Gfap (**K**), S100β (**L**), and DAPI in shCtrl and shMysm1 NSCs under undifferentiated and differentiated conditions. Percentage (right) of Gfap^+^ (**K**) or S100β^+^ (**L**) cells among total shCtrl and shMysm1 NSCs (*n* = 5). Data are presented as the mean value ± SEM. ns, not significant; *P < 0.05*,* **P < 0.01*,* ***P < 0.001, and ****P < 0.0001. Scale bar represents 20 μm (**D**, **E**, **F**, **K** and **L**).

To further study whether Mysm1 regulates the differentiation of NSCs, NSCs were isolated and transduced with shCtrl or shMysm1 lentivirus and then induced for neuron, astrocyte or oligodendrocyte differentiation (Fig. 3A, H and Supplementary Fig. 3G). qRT-PCR analysis showed that cultures of Mysm1 knockdown NSCs exhibited increased neuronal markers Dlx1, Gad67, and Tbr2 expression compared to control NSCs (Fig. 3B). Western blot analysis was used to detect the expression of neuron-related proteins in shCtrl and shMysm1 NSCs induced for 3 days. The results showed that the expression of Map2 and Dcx was higher in shMysm1 NSCs than in shCtrl NSCs (Fig. 3C). There were no significant changes after knocking down Mysm1 under undifferentiated conditions, but under 2-day or 4-day differentiated conditions, there was a significant increase in Map2^+^ cells (Fig. 3D) and β3-tubulin^+^ cells (Fig. 3E), a robust decrease in Nestin^+^ cells (Supplementary Fig. 3A) and Ki67^+^ cells in shMysm1 NSCs compared to shCtrl NSCs (Supplementary Fig. 3B). We induced shCtrl and shMysm1 NSCs to differentiate into neurons for 7 days. After reconstructing and visualizing the dendrites, we compared the length and number of dendrites by Sholl analysis. The results showed that the number of dendrites was greater and the dendrite length was longer in shMysm1 NSCs compared to shCtrl NSCs (Fig. 3F). At the same time, a multielectrode array system was used to examine the electrophysiological activity, and the average spikes of each active electrode per minute were higher in shMysm1 NSCs compared to shCtrl NSCs (Fig. 3G). A significant increase in the TH and VGLUT2 functional neuronal markers was also observed in shMysm1 NSCs compared to controls after induction for 7 days (Supplementary Fig. 3C, D).

In view of the significant difference in neuron differentiation, we induced shCtrl and shMysm1 NSCs to differentiate into astrocytes. qRT-PCR analysis showed that Mysm1 knockdown NSCs exhibited decreased Notch1, Hes5, and S100β expression compared to controls (Fig. 3I). Western blot analysis was used to detect the expression of astrocyte-related proteins in shCtrl and shMysm1 NSCs induced for 3 days. The results showed that the expression of Gfap and Aldh1l1 was lower in shMysm1 NSCs than in shCtrl NSCs (Fig. 3J). Under 2-day or 4-day differentiated conditions, we observed a significant decrease in Gfap^+^ cells and S100β^+^ cells (Fig. 3K, L) as well as a significant increase in Nestin^+^ cells (Supplementary Fig. 3E) in shMysm1 NSCs compared to shCtrl NSCs. Ki67^+^ cells remained elevated as well as undifferentiated in shMysm1 NSCs compared to shCtrl NSCs (Supplementary Fig. 3F).

We also induced shCtrl and shMysm1 NSCs to differentiate into oligodendrocytes. Sox10 and Olig2 are transcription factors specific to oligodendrogenesis [28]. qRT-PCR analysis showed that Mysm1 knockdown NSCs exhibited decreased Sox10 expression compared to controls, but the expression of Olig2 did not change significantly (Supplementary Fig. 3H). Western blot analysis was used to detect the expression of oligodendrocyte-related proteins in shCtrl and shMysm1 NSCs induced for 7 days. The results showed that Olig2 and NG2 were expressed similarly between shMysm1 and shCtrl NSCs (Supplementary Fig. 3I). Under 7-day differentiated conditions, we observed no significant difference in the percentages of Olig2^+^ cells and NG2^+^ cells between shMysm1 NSCs and shCtrl NSCs (Supplementary Fig. 3J, K).

These results indicate that Mysm1 is involved in regulating the differentiation of NSCs in vitro and that Mysm1-deficient NSCs are more likely to commit to neuronal lineages.

### Mysm1 regulates the transcription of Id4

**Fig. 4:**
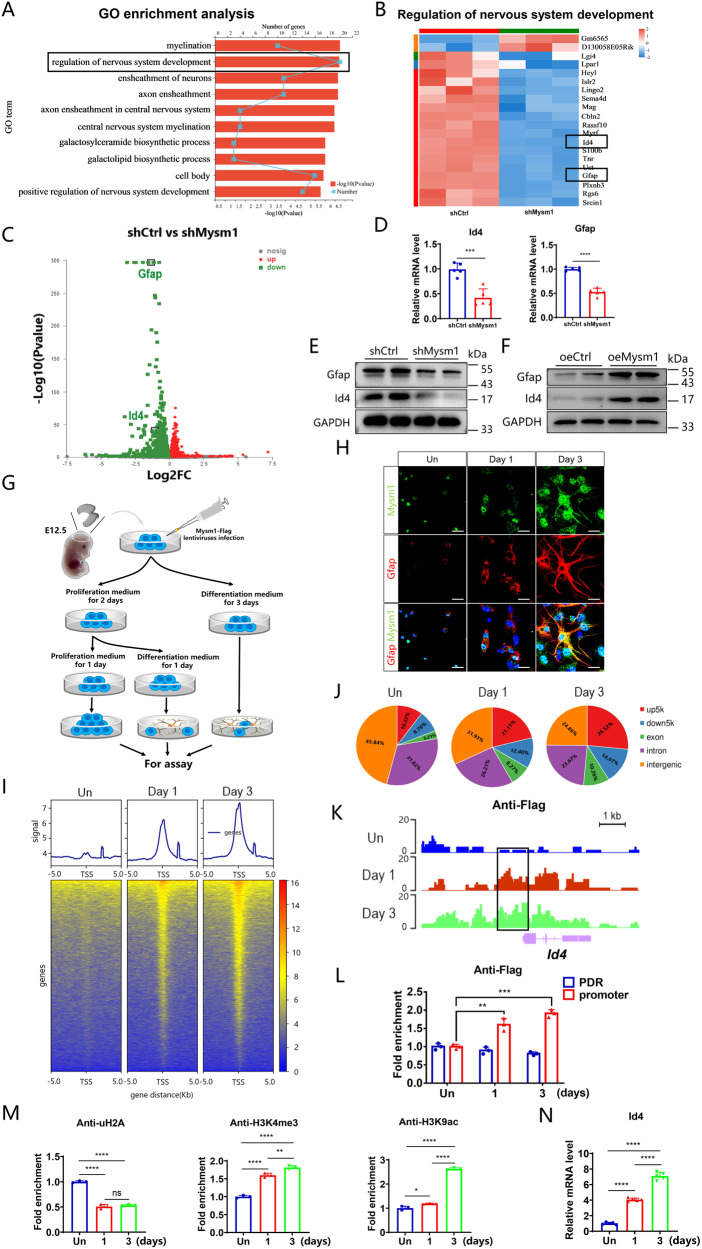
Mysm1 epigenetically regulates the transcription of Id4. **A** GO enrichment gene number is defined as the number of target genes in each term. The rich factor is defined as the number of target genes divided by the number of all the genes in each term. The number of GO target genes, *p* value, and rich factor are indicated in the column chart with broken lines. **B** Cluster heatmap of representative differentially expressed genes in shMysm1 and shCtrl NSCs. Red indicates upregulation, and blue indicates downregulation. **C** Volcano maps of differentially expressed genes in shMysm1 and shCtrl NSCs. Red indicates upregulation, and green indicates downregulation. qRT-PCR (**D**, *n* = 5) and western blot (**E**) analyses verified the lower levels of Id4 and Gfap in shMysm1 NSCs compared to shCtrl NSCs. **F** Western blot analysis verified the higher levels of Id4 and Gfap in oeMysm1 NSCs compared to oeCtrl NSCs. **G** Schematic diagram of virus infection and induction of NSCs to differentiate into astrocytes. **H** Immunofluorescence for Mysm1, Gfap, and DAPI in NSCs under undifferentiated and differentiated conditions. **I** The binding density of Flag was visualized by deepTools. The heatmap presents the CUT&Tag tag counts on the different Flag-binding peaks in NSCs in undifferentiation and differentiation conditions (ordered by signal strength). **J** Genome-wide distribution of upregulated Flag-binding peaks in NSCs under undifferentiated and differentiated conditions. **K** Genome browser tracks CUT&Tag signal at the representative target gene loci. The box indicates the peak regions of Flag on the Id4 promoter region. **L** CUT&RUN-qPCR was used to detect the enrichment of Flag on the Id4 promoter region or promoter-deprived region (PDR) in undifferentiated and differentiated conditions (*n* = 3). **M** CUT&RUN-qPCR was used to detect the enrichment of the indicated proteins on the Id4 promoter region under undifferentiated and differentiated conditions (*n* = 3). **N** qRT-PCR analysis of the relative mRNA levels of Id4 in NSCs under undifferentiated and differentiated conditions (*n* = 5). Histone H3 lysine 4 trimethylation, H3K4me3; histone H3 lysine 9 acetylation, H3K9ac; ubiquitin-histone H2A, uH2A. Data are presented as the mean value ± SEM. ns not significant; *P < 0.05*,* **P < 0.01*,* ***P < 0.001, and ****P < 0.0001. Scale bar represents 20 μm (**H**).

To explore the mechanism by which Mysm1 regulates the function of NSCs, transcriptomic analysis using an Affymetrix Clariom D array was performed to examine the transcripts in shCtrl and shMysm1 NSCs. GO functional enrichment analysis demonstrated that the number and degree of differentially expressed genes enriched in the function of “regulation of nervous system development” were significant after knocking down Mysm1 (Fig. 4A). Heatmap assays of differentially expressed genes in nervous system developmental regulation identified differences in gene expression between shCtrl and shMysm1 NSCs (Fig. 4B). KEGG enrichment analysis also demonstrated differences in embryonic development, tissue and organ formation, and adult homeostasis-related pathways such as the TGF-β pathway (Supplementary Fig. 4A, B). Previous studies have shown that inhibitors of DNA-binding factor 4 (Id4) and Gfap are involved in the development of astrocytes [29–32]. Interestingly, the heatmap indicated that Gfap and Id4 had extremely low expression. The volcano map showed that some genes were significantly upregulated and downregulated. The expression of Gfap and Id4 was significantly decreased (Fig. 4C), which was further confirmed in shMysm1 NSCs by qRT-PCR (Fig. 4D) and western blot analyses (Fig. 4E). We then used a lentivirus expressing Mysm1 (oeMysm1) to overexpress Mysm1 in NSCs, and the expression of Id4 and Gfap was detected by western blot analysis. In oeMysm1 NSCs, the Id4 and Gfap protein levels were significantly increased compared to control NSCs (Fig. 4F). Thus, these data indicated that Mysm1 may regulate the transcription of Id4 and Gfap.

Because Mysm1 is a transcriptional regulator, we next investigated how Mysm1 regulates Id4 and Gfap using genome-wide CUT&Tag analysis to detect the recruitment of Mysm1 at the promoter region of genes. NSCs were first transduced with lentivirus expressing Mysm1 and a Flag tag. NSCs were then induced to proliferate and undergo astrocyte differentiation for 1 day and 3 days (Fig. 4G). Immunofluorescence analysis indicated costaining of Mysm1 and Gfap (Fig. 4H). Western blot and real-time PCR analyses demonstrated that the expression of the Id3 and Gfap astrocyte markers increased with increasing induction time (Supplementary Fig. 4C, D) but that the relative mRNA level of the Tbr2 neuronal lineage intermediate progenitor cell marker decreased (Supplementary Fig. 4E). CUT&Tag using antibodies against Flag and analysis with deepTools revealed a gradual enhanced enrichment of Flag peaks in NSCs that stably expressed Mysm1-Flag with increasing induction time (Fig. 4I). Moreover, the distribution of Flag-binding peaks in the whole genome indicated a certain percentage of enrichment peaks in the promoter sequences (up5k) (Fig. 4J), and the called peaks showed that the recruitment level of Flag, which indicating enrichment of Mysm1, was gradually elevated on the Id4 gene locus with increasing induction time (Fig. 4K). By comparing the conserved regions in the Id4 promoter sequence of different species with mice, we found that the Flag signal peak of the Id4 promoter region coincided with the conserved regions, and we designed primers from the boxed promoter region and promoter-deprived region (PDR) (Supplementary Fig. 4F). We then performed CUT&RUN-qPCR analysis. Compared to the enrichment on PDR, the Flag levels on the Id4 promoter were significantly elevated with increasing induction time (Fig. 4L), and the histone H3 lysine 4 trimethylation (H3K4me3) and histone H3 lysine 9 acetylation (H3K9ac) levels on the Id4 promoter were also significantly elevated; however, the levels of ubiquityl-Histone H2A (uH2A) were decreased (Fig. 4M). Consistently, qRT-PCR analysis indicated that the expression of Id4 gradually increased with increasing induction time (Fig. 4N). Collectively, these data revealed that Mysm1 orchestrates histone modifications at the Id4 promoter region and activates Id4 transcription.

### Overexpression of Id4 partially restores the function of Mysm1-deficient NSCs

**Fig. 5:**
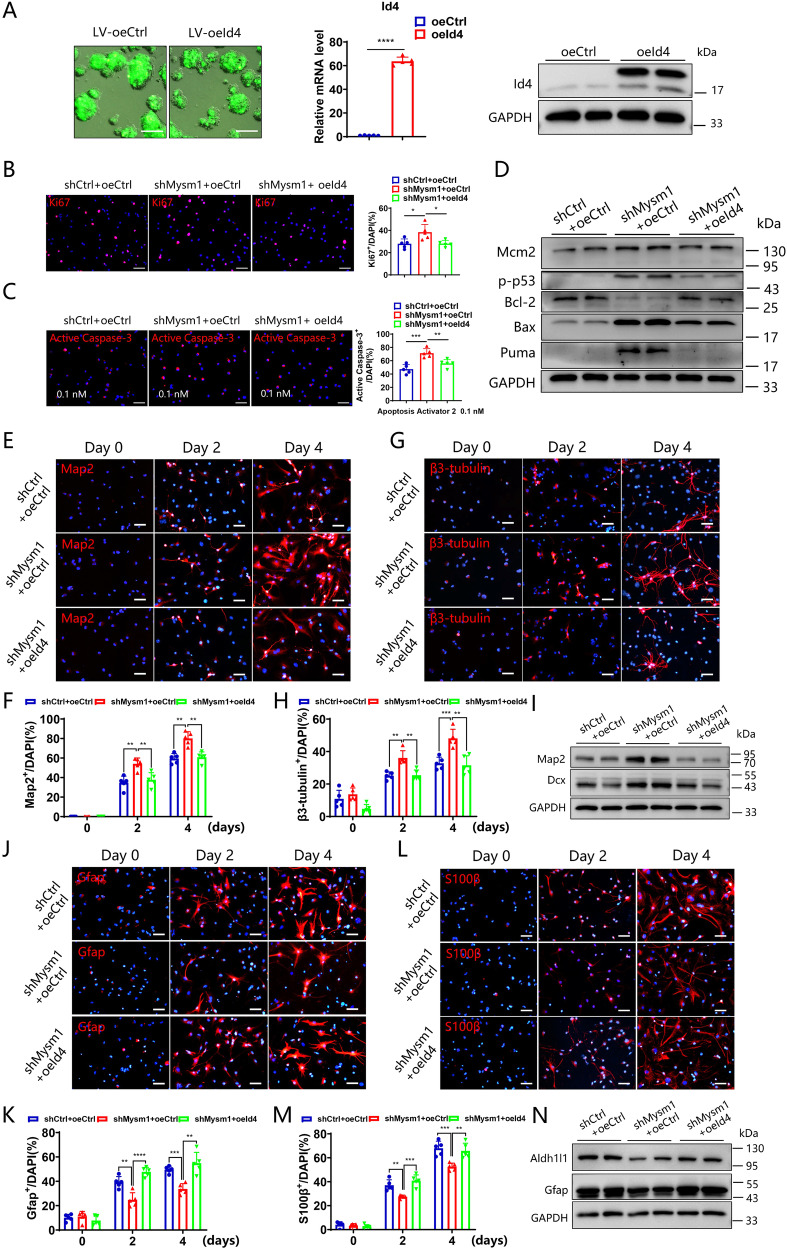
Overexpression of Id4 partially restores the function of Mysm1-deficient NSCs. **A** After transduction of WT NSCs with overexpressing control and Id4- overexpressing lentiviruses, the overexpression efficiency was verified by green fluorescence (left), qRT-PCR analysis (middle, *n* = 5) and western blot analysis (right). **B** Immunofluorescence (left) for Ki67 and DAPI in control NSCs and shMysm1 NSCs before and after Id4 overexpression. Percentage (right) of Ki67^+^ NSCs among total control NSCs and shMysm1 NSCs before and after Id4 overexpression (*n* = 5). **C** Immunofluorescence (left) for Active Caspase-3 and DAPI in control NSCs and shMysm1 NSCs before and after Id4 overexpression followed by treatment with 0.1 nM Apoptosis Activator 2. Percentage (right) of Active Caspase-3^+^ cells among total control NSCs and shMysm1 NSCs before and after Id4 overexpression (*n* = 5). **D** Western blot analysis of the indicated proteins in control NSCs and shMysm1 NSCs before and after Id4 overexpression. Immunofluorescence (upper) for Map2 (**E**), β3-tubulin (**G**), and DAPI in control NSCs and shMysm1 NSCs before and after Id4 overexpression under undifferentiated and differentiated conditions. Percentage (lower) of Map2^+^ (**F**) or β3-tubulin^+^ (**H**) cells among total control NSCs and shMysm1 NSCs before and after Id4 overexpression (*n* = 5). **I** Western blot analysis of the indicated proteins in control NSCs and shMysm1 NSCs before and after Id4 overexpression after 3 days of differentiation into neurons. Immunofluorescence (upper) analysis of Gfap (**J**), S100β (**L**), and DAPI in control NSCs and shMysm1 NSCs before and after Id4 overexpression under undifferentiated and differentiated conditions. Percentage (lower) of Gfap^+^ (**K**) or S100β^+^ (**M**) cells among total control NSCs and shMysm1 NSCs before and after Id4 overexpression (*n* = *5*). **N** Western blot analysis of the indicated proteins in control NSCs and shMysm1 NSCs before and after Id4 overexpression after 3 days of differentiation into astrocytes. Data are presented as the mean value ± SEM. *p < 0.05*,* **p < 0.01*,* ***p < 0.001, and ****P < 0.0001. Scale bars represent 50 μm (**A**) and 20 μm (**B**, **C**, **E**, **G**, **J**, and **L**).

Considering that Mysm1 regulates Id4 transcription and the decreased expression of Id4 in shMysm1 NSCs, we hypothesized that overexpression of Id4 in shMysm1 NSCs would partially rescue the impaired function of NSCs. A lentivirus expressing Id4 and green fluorescent protein (oeId4) was generated to transduce NSCs. Green fluorescence was significant in the neurospheres, indicating successful transduction. qRT-PCR and western blot analyses showed that the expression of Id4 was significantly higher in NSCs transduced with oeId4 than in NSCs transduced with control virus (oeCtrl), which suggested the successful overexpression of Id4 in NSCs (Fig. 5A). shMysm1 NSCs were then transduced with oeId4 or control lentivirus, and Ki67 staining was performed. As shown in Fig. 5B, the higher proportion of Ki67^+^ cells in shMysm1 NSCs was reversed by Id4 overexpression. To detect the change in apoptosis, control NSCs, shMysm1 NSCs with or without Id4 overexpression were treated with 0.1 nM Apoptosis Activator 2. The results showed that the percentage of Active Caspase-3^+^ cells in shMysm1 NSCs with Id4 overexpression was significantly decreased (Fig. 5C). Similarly, western blot analysis showed that the expression of Mcm2, p-p53, Bax, and Puma was decreased but that the expression of Bcl-2 was increased after Id4 restoration (Fig. 5D). Control NSCs and shMysm1 NSCs with or without Id4 overexpression were then induced to undergo neural differentiation for 2 or 4 days. There was a significant decrease in the proportion of Map2^+^ cells and β3-tubulin^+^ cells after Id4 restoration (Fig. 5E–H). Western blot analysis detected decreased expression of the Map2 and Dcx neuron-related markers, which confirmed the suppressed neural differentiation of neurons promoted by Id4 restoration (Fig. 5I). In addition to neural differentiation, control NSCs and shMysm1 NSCs with or without Id4 overexpression were induced to undergo astrocyte differentiation. There was a significant increase in the proportion of Gfap^+^ and S100β^+^ cells in shMysm1 NSCs overexpressing Id4 (Fig. 5J–M). Western blot analysis showed that after restoration of Id4, the expression of the astrocyte-related markers, Gfap and Aldh1l1, was increased under astrocyte differentiation conditions for 3 days (Fig. 5N). These results revealed that exogenous expression of Id4 rescues some characteristics and functions in Mysm1-deficient NSCs.

### Mysm1 knockdown promotes NSC proliferation in the brains of aged mice

**Fig. 6:**
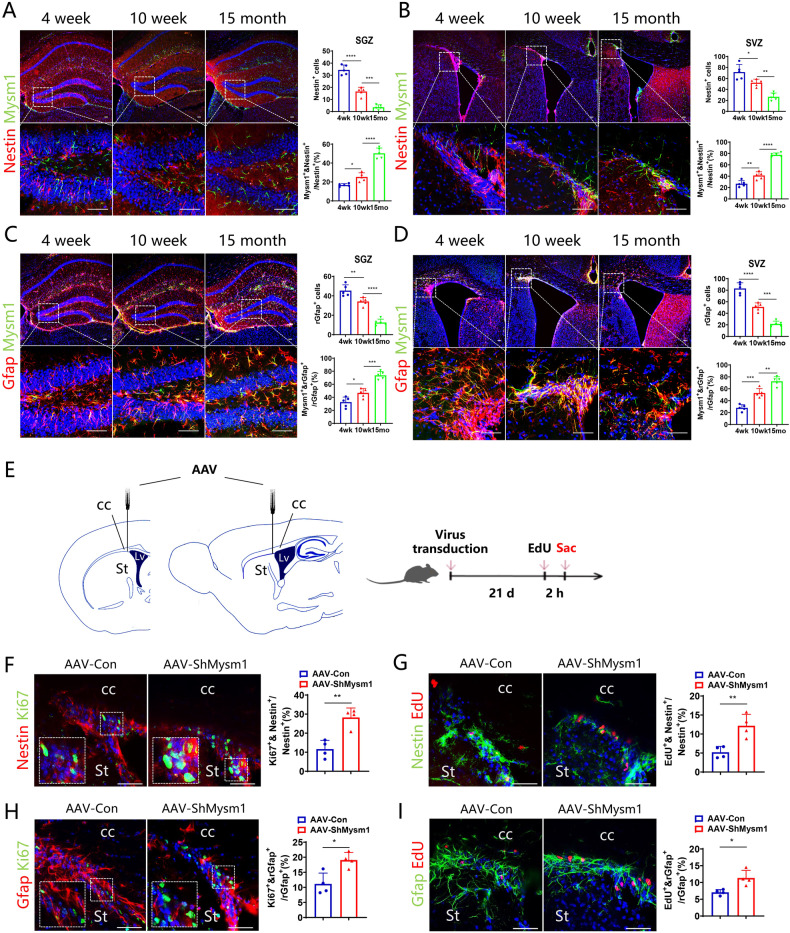
Mysm1 knockdown promotes NSC proliferation in the brains of aged mice. Immunofluorescence (left) for Nestin and Mysm1 in the SGZ (**A**) or SVZ (**B**) of 4-week-old, 10-week-old, and 15-month-old WT mice. The boxed areas are shown in detail at the bottom. Percentage (right) of Mysm1^+^ Nestin^+^ NSCs among total Nestin^+^ NSCs per SGZ (**A**) or SVZ (B) section of 4-week-old, 10-week-old, and 15-month-old WT mice (*n* = 5). Immunofluorescence (left) for Gfap and Mysm1 in the SGZ (**C**) or SVZ (**D**) of 4-week-old, 10-week-old, and 15-month-old WT mice. The boxed areas are shown in detail at the bottom. Percentage (right) of Mysm1^+^ rGfap^+^ NSCs among total rGfap^+^ NSCs per SGZ (**C**) or SVZ (**D**) section of 4-week-old, 10-week-old, and 15-month-old WT mice (*n* = 5). **E** Schematic of coronal and sagittal sections showing the location of AAV injection in the SVZ. Brains were analyzed 2 h after EdU injection. **F** Immunofluorescence (left) for Nestin and Ki67 in the SVZ of AAV-Con and AAV-ShMysm1 mice. The boxed areas are shown in detail as insets. Percentage (right) of Ki67^+^ Nestin^+^ NSCs among total Nestin^+^ NSCs per SVZ section of AAV-Con and AAV-ShMysm1 mice (*n* = 4). **G** Immunofluorescence (left) for Nestin and EdU in the SVZ of AAV-Con and AAV-ShMysm1 mice. Percentage (right) of EdU^+^ Nestin^+^ NSCs among total Nestin^+^ NSCs per SVZ section of AAV-Con and AAV-ShMysm1 mice (*n* = 4). **H** Immunofluorescence (left) for Gfap and Ki67 in the SVZ of AAV-Con and AAV-ShMysm1 mice. The boxed areas are shown in detail as insets. Percentage (right) of Ki67^+^ rGfap^+^ NSCs among total rGfap^+^ NSCs per SVZ section of AAV-Con and AAV-ShMysm1 mice (*n* = 4). **I** Immunofluorescence (left) for Gfap and EdU in the SVZ of AAV-Con and AAV-ShMysm1 mice. Percentage (right) of EdU^+^ rGfap^+^ NSCs among total rGfap^+^ NSCs per SVZ section of AAV-Con and AAV-ShMysm1 mice (*n* = 4). Data are presented as the mean value ± SEM. *p < 0.05*,* **p < 0.01, ***p < 0.001, and ****P < 0.0001. Scale bar represents 50 μm (**A**, **B**, **C**, **D**, **F**, **G**, **H**, and **I**).

Considering the hyperproliferation of NSCs with Mysm1 deficiency, we wondered whether knocking down Mysm1 in NSCs from aged brain would promote NSC proliferation. To clarify the relationship between the abundance of Mysm1 in NSCs and age, we performed immunofluorescence staining. The number of Nestin^+^ NSCs or rGfap^+^ NSCs decreased in the SGZ and SVZ with increasing age, while the proportion of Mysm1^+^ cells in Nestin^+^ NSCs or rGfap^+^ NSCs increased, which suggested that the expression of Mysm1 in NSCs in the mouse brain accumulated with age (Fig. 6A–D). To evaluate the changes in the proliferation ability of NSCs with age, Ki67^+^ cells in these Nestin^+^ NSCs or rGfap^+^ NSCs in the SGZ and SVZ were counted. The proportion of Ki67^+^ cells decreased significantly with mouse age (Supplementary Fig. 5A-D). These results suggested that the proliferation ability of NSCs gradually decreased with age. To confirm whether the acquired knockdown of Mysm1 in vivo has the similar effect, we knocked down Mysm1 in Gfap^+^ NSCs. An AAV expressing Mysm1 shRNA (AAV-ShMysm1), which interferes with the Mysm1 sequence under the Gfap promoter, was directly injected into the SVZ of 14-month-old WT mice, and the proliferation ability of NSCs in vivo was detected after 3 weeks (Fig. 6E). The results showed that the proportion of Ki67^+^ Nestin^+^ NSCs or EdU^+^ Nestin^+^ NSCs to Nestin^+^ NSCs in the SVZ of AAV-ShMysm1 mice was significantly increased compared to controls (AAV-Con) (Fig. 6F, G), and the proportion of Ki67^+^ rGfap^+^ NSCs or EdU^+^ rGfap^+^ NSCs to rGfap^+^ NSCs was significantly increased compared to AAV-Con mice (Fig. 6H, I). These results revealed that Mysm1 knockdown in NSCs of aged mice promoted NSC proliferation in vivo.

## Discussion

**Fig. 7:**
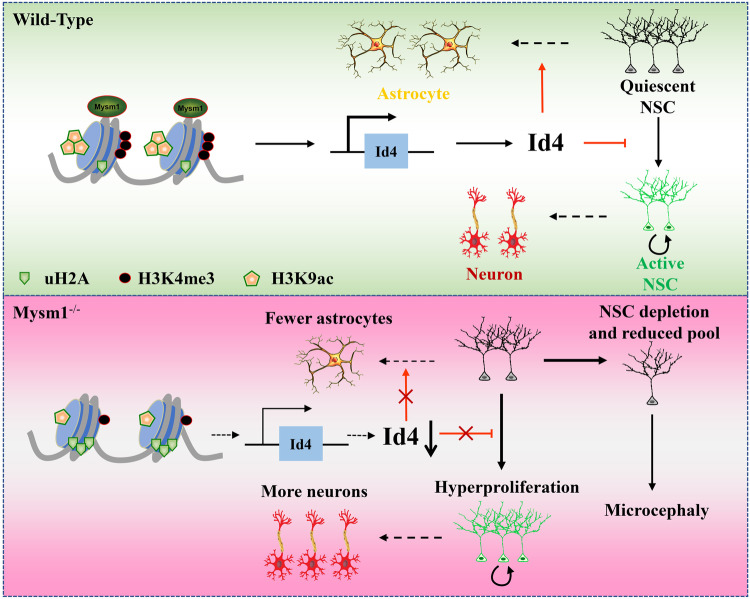
Schematic model for the function of Mysm1 in regulating neural stem cell proliferation and differentiation. Mysm1 regulates NSC proliferation and differentiation by controlling the transcription of Id4. Mysm1-deficient NSCs exhibited hyperproliferation, enhanced neurogenesis, and compromised astrogliogenesis.

To the best of our knowledge, the present study demonstrated for the first time that Mysm1 knockout in NSCs can lead to phenotypic changes in the brain. The data revealed that Mysm1-deficient NSCs exhibited hyperproliferation, increased apoptosis, enhanced neurogenesis, and compromised astrogliogenesis. Moreover, Id4 overexpression in Mysm1-deficient NSCs partially reversed the dysfunction of NSCs (Fig. 7).

Mysm1 has been reported to play important roles in many cells, such as HSCs, MSCs, immune cells, and astrocytes [13, 14, 16, 19, 21]. Here, we found that Mysm1 was expressed in Nestin-positive NSCs. Mice with Mysm1 conditional knockout in NSCs showed abnormal brain development. Mysm1 deficient NSCs exhibit hyperproliferation both in vivo and in vitro. There was no difference in the expression of apoptosis markers in NSCs before and after knockout of Mysm1 in vivo, but treatment of Mysm1-deficient cells with Apoptosis Activator 2 in vitro led to increased expression of apoptosis markers in NSCs and decreased cell viability. These results indicated that Mysm1 protects NSCs from apoptosis under certain stresses. P53 is one of the central regulators of the cellular stress response [33–35]. Our western blot analysis demonstrated that Mysm1-deficient NSCs showed increased p53 phosphorylation but with no significant changes in total p53 in vitro. In addition, the Bax and Puma apoptotic proteins downstream of p53 were also increased in Mysm1-deficient NSCs, while the anti-apoptotic protein, Bcl-2, was decreased in Mysm1-deficient NSCs. Studies have shown that Mysm1 plays an important role in bone marrow progenitor cells, lymphoid progenitor cells, developing thymocytes, and other types of cells to protect against p53-mediated apoptosis. [13, 33, 36–40] Hyperactivation of p53 due to Mysm1 deficiency leads to apoptosis of multipotent progenitor cells and promotes the loss of the quiescent state of HSCs [40]. The knockdown of Mysm1 shifts HSCs from a quiescent state to a rapid cycle and increases their apoptosis rate, which leads to the depletion of the stem cell pool and impairs the self-renewal and lineage reconstruction abilities of Mysm1-deficient mice [13]. Mysm1 deficiency also reduces the colony formation of epidermal progenitor cells, and p53 is a potential mediator of defects, resulting in increased expression of proapoptotic and antiproliferative genes [39]. Our data and other studies [33, 37, 40] indicate that Mysm1 regulates homeostasis of different stem cells such as NSCs, HSCs partly by similar p53 signaling pathway.

Regarding the regulation of NSC differentiation by Mysm1, we found that Dcx^+^ cells in the DG of Mysm1 cKO mice increased at 6 and 9 weeks of age compared to CTRL mice. In addition, we also observed the developmental disturbance of astrocytes in Mysm1 cKO mice. In vitro experiments also confirmed that NSCs with Mysm1 knockdown induced more neuron-like cells and fewer astrocyte-like cells. These results indicated that Mysm1 plays an important role in controlling the differentiation balance of NSCs. Some studies have shown that Id4 maintains the quiescent state of NSCs, while the increased expression of Id4 leads to glial formation together with less neurogenesis. In addition, Id4 knockdown leads to a decrease in astrocytes [29, 41]. During the differentiation of NSCs into astrocytes, Id4 expression was induced, and the enrichment of Mysm1 in the Id4 promoter region was significantly increased. At the same time, the uH2A level on the Id4 promoter was significantly decreased, and the H3K4me3 and H3K9ac levels were also significantly elevated. Previous studies have revealed that ubiquitination of histone H2A is involved in gene repression [42, 43]. Zhu et al. have showed that Mysm1 participate in transcriptional initiation and possibly also in elongation, and a series of distinct histone-modifying coactivators are included to remove repressive marks (trimethylated histone H3 at lysine 27 (H3K27me3), uH2A, etc.) and add active marks (H3K9ac, H3K4me, etc.) to achieve the “optimal” modulation of nucleosome architecture for transcriptional activation [11]. Several other studies have shown the function of Mysm1 in regulating the ubiquitin modification of histone H2AK119 (histone H2A lysine 119), accompanied by changes in methylation and acetylation modifications in the promoter region of transcription factors, thereby altering the chromatin structure of the promoter region to facilitate transcription factor expression [12, 13, 19]. In B cell precursors, Mysm1 selectively removes uH2A at the Ebf1 locus to regulate cell development [12]. Mysm1 regulates self-renewal and differentiation of HSCs by regulating Gfi1 expression. After Mysm1 knockout, the dynamic balance of HSCs is disrupted, leading to depletion of stem cell pool [13]. Mysm1 also regulates the expression of Id2 to regulate natural killer cell development [19]. In NSCs, we have observed a similar situation, indicating that Mysm1 orchestrates histone modifications and activates Id4 transcription. Further research is required on how Mysm1 cooperates with other protein complexes in regulating the histone modifications at the promoter region of Id4. Previous studies have demonstrated the role of Id4 in inhibiting the activation of NSCs. Studies have shown that Id4 is highly expressed in quiescent hippocampal stem cells [44]. Thus, we speculate that the decrease in Id4 caused by knocking down Mysm1 may account for the overactivation and decreased glial formation of NSCs.

The regulation of stem cell fate is highly complex. In the nervous system, many factors affect the activity and cell fate of NSCs. Previous studies have suggested that different levels of Ascl1 lead to three possible states of NSCs, namely, quiescence, proliferation, and differentiation, which correspond to a lack of Ascl1 expression, low Ascl1 expression levels, and high Ascl1 expression levels, respectively [45]. Notch signaling is the central regulator of NSC fate and plays a key role in regulating maintenance, proliferation, and differentiation [46–48]. Notch2 regulates the expression of the Hes gene, while Hes1 and Hes5 inhibit the expression of the Ascl1 gene [41]. In addition, Notch2 has been reported to maintain the quiescent state of V-SVZ NSCs and Notch2-Id4 axis promotes NSC quiescence [49]. Our data indicated that Mysm1-Id4 axis is also important for NSC fate decision.

Multiple genes/pathways have been demonstrated to be associated with maintenance of quiescence, such as Clu [50], Hopx [51], Notch2 [49], and Id4 [44]. The reduced expression of quiescence-associated genes maintains resting NSCs in shallow quiescence, which allows them to reactivate much more readily than dormant cells and sustain NSC proliferation and neurogenesis over the long term [52]. We found that Mysm1 was enriched in NSCs from aged animals. Aged animals have significantly lower NSC numbers, and most of them are quiescent cells. The accumulative expression of Mysm1 may be associated with the lower proliferation of NSCs from aged brains. In aged patients, Mysm1 is highly expressed in the SGZ and SVZ, which are NSC niches. Considering the hyperproliferative property of Mysm1 deficient NSCs, knockdown Mysm1 in NSCs from aged brains may be an ideal strategy for inducing NSC proliferation, and thereby promoting neurogenesis. Neurogenesis is believed to be an important neurorestorative mechanism [53], the present study revealed a new factor may be a valuable target for brain disorder therapy.

In summary, the present study identified Mysm1 as a novel factor essential for NSC homeostasis and the Mysm1-Id4 axis may be an ideal target for proper NSC proliferation and differentiation.

### Supplementary information

Supplementary information

Supplementary Figure 1

Supplementary Figure 2

Supplementary Figure 3

Supplementary Figure 4

Supplementary Figure 5

Supplementary Figure 6

Original western blots

## Data Availability

The raw data supporting the findings of this study will be made available by the corresponding authors upon reasonable request.
