# Supplementary material for: Deubiquitinase Mysm1 regulates neural stem cell proliferation and differentiation by controlling Id4 expression
Source: Cell Death Dis. 2024 Feb 12;15(2):129. doi: 10.1038/s41419-024-06530-y (PMC10859383; doi:10.1038/s41419-024-06530-y)
Supplement: Supplementary file 1 — Supplementary information [file 41419_2024_6530_MOESM1_ESM.docx]

**Supplementary figures**

**Supplementary Figure 1 Mysm1 deletion in NSCs leads to aberrant brain development**

(A) The sagittal and coronal brain slices of 10-month-old mice were compared. The dotted lines on the sagittal slices correspond to the coronal slices in the middle. The boxed areas are shown in detail on the right side. (B, C) Immunofluorescence (left) for Gfap and DAPI in the DG of 6-week-old (B) and 9-week-old (C) CTRL and Mysm1 cKO mice. The boxed areas are shown in detail at the bottom. Quantification (right) of the average optical density of Gfap in the DG of 6-week-old (B) and 9-week-old (C) CTRL and Mysm1 cKO mice (*n=3*). Data are presented as the mean value ± SEM. **p < 0.05.* Scale bars represent 1 mm (A) and 100 μm (B andC).

**Supplementary Figure 2 Mysm1 knockdown promotes the proliferation and apoptosis of NSCs in vitro**

shCtrl and shMysm1 NSCs were separated by serial dilution, and neurosphere formation was induced for 7 days at a density of 1 × 10^5^ cells/ml in vitro. The relative size of the first- and second-generation spheres grown for 7 days was quantified by ImageJ software. Data are presented as the mean value ± SEM. *ns, not significant; *p < 0.05, **p < 0.01, and ****P < 0.0001*. Scale bar represents 20 μm.

**Supplementary Figure 3 Mysm1 knockdown skews the differentiation of NSCs in vitro**

(A, B) Immunofluorescence (left) for samples equivalent to those shown in Figure 3D with Nestin (A), Ki67 (B), and DAPI staining. Quantification (right) of Nestin^+^ (A) or Ki67^+^ (B) cells in samples analyzed on the left (n = 5). (C, D) Immunofluorescence (left) for TH (C), VGLUT2 (D), and DAPI in shCtrl and shMysm1 NSCs after 7 days of differentiation into neurons. Quantification (right) of TH^+^ (C) or VGLUT2^+^ (D) cells in samples analyzed on the left (n = 5). (E, F) Immunofluorescence (left) for samples equivalent to those shown in Figure 3K with Nestin (E), Ki67 (F), and DAPI. Quantification (right) of Nestin^+^ (E) or Ki67^+^ (F) cells in samples analyzed on the left (n = 5). (G) Time schedule for the in vitro culture of neurospheres and induction of NSCs into oligodendrocytes, experiments at different time points are marked on the timeline. (H) qRT-PCR analysis of the indicated transcripts in shCtrl and shMysm1 NSCs (n=3). (I) Western blot analysis (left) of the indicated proteins in shCtrl and shMysm1 NSCs after 7 days of differentiation into oligodendrocytes, and the protein level was normalized to GAPDH (right, n=3). (J, K) Immunofluorescence for Olig2 (J, left), NG2 (K), and DAPI in shCtrl and shMysm1 NSCs under differentiated conditions. Percentage (J, right) of Olig2^+^ cells among total cells of shCtrl and shMysm1 NSCs (n=5). Data are presented as the mean value ± SEM. *ns, not significant; *p < 0.05, **p < 0.01 and ***p < 0.001.* Scale bar represents 20 μm (A, B, C, D, E, F, J and K).

**Supplementary Figure 4 Mysm1-Flag is enriched on the target loci of some genes**

(A) KEGG enrichment gene number is defined as the number of target genes in each term. The rich factor is defined as the number of target genes divided by the number of all the genes in each term. The number of GO target genes, p value, and rich factor are indicated in the column chart with broken lines. (B) Cluster heatmap of representative differentially expressed genes in shMysm1 and shCtrl NSCs. Red indicates upregulation, and blue indicates downregulation. (C) Western blot analysis showing the expression and Gfap in NSCs under undifferentiated and differentiated conditions. (D, E) qRT-PCR analysis of the relative mRNA levels of Id3 (D, left), Gfap (D, right), and Tbr2 (E) in NSCs under undifferentiated and differentiated conditions (*n=5*). (F) Screenshots of the conservation track of Id4 locus in the mouse UCSC Genome Browser. The conservation track has two parts as follows: a plot of conservation scores, and a display showing where each of the other genomes aligns to the reference genome (darker shading indicates higher BLASTZ scores, and white indicates no alignment). The sequences of the promoter region and PDR were used to design primers. PDR, promoter-deprived region. Data are presented as the mean value ± SEM. *ns, not significant*; ****P < 0.001,* and *****P < 0.0001*.

**Supplementary Figure 5 Proliferation ability of NSCs decrease with age**

(A, B) Immunofluorescence (left) for Nestin and Ki67 in the SGZ (A) or SVZ (B) of 4-week-old, 10-week-old, and 15-month-old WT mice. The boxed areas are shown in detail at the bottom. Percentage (right) of Mysm1^+^ Ki67^+^ NSCs among total Nestin^+^ NSCs per SGZ (A) or SVZ (B) section of WT mice at 4 weeks, 10 weeks, and 15 months (*n=5*). (C, D) Immunofluorescence (left) for Gfap and Ki67 in the SGZ (C) or SVZ (D) of 4-week-old, 10-week-old, and 15-month-old WT mice. The boxed areas are shown in detail at the bottom. Percentage (right) of Ki67^+^ rGfap^+^ NSCs among total rGfap^+^ NSCs per SGZ (C) or SVZ (D) section of WT mice at 4 weeks, 10 weeks, and 15 months (*n=5*). Data are presented as the mean value ± SEM. *ns, not significant; **p < 0.01* and *****P < 0.0001*. Scale bar represents 50 μm (A, B, C and D).

**Supplementary Figure 6** Diagrams of the LV and AAV vectors used in the studies.

**Supplementary tables**

| **Supplementary table 1 Sequences of primers used for genotype identification** | | |
| --- | --- | --- |
| **Name** | **Forward (5’-3’)** | **Reversed (5’-3’)** |
| Mysm1 | GGGGATGGAAGAGAAAGGAAAGAATCCAC | AGTCTGTTTTTCTTCGGCCAGAACCTGAG |
| Cre | GCCTGCATTACCGGTCGATGC | CAGGGTGTTATAAGCAATCCC |

| **Supplementary table 2 Sequences of primers used for RT - qPCR analysis of mRNA levels** | | |
| --- | --- | --- |
| **Name** | **Forward (5’-3’)** | **Reversed (5’-3’)** |
| β-actin | GGCTGTATTCCCCTCCATCG | CCAGGTAACAATGCCATG |
| Tbr2 | ATTGTCCCTGGAGGTCGGTACG | GTTGGTCTGTGGCACGGTTCTC |
| Mysm1 | AAGCACCGTTAGCCTCTTCGTTTC | CCTTCCGTCAGGACTCAGCAATG |
| Gfap | AGATTCGCACTCAATACGAGG | CTGTGAGGTCTGCAAACTTAGA |
| S100β | TTGATGTCTTCCACCAGTACTC | CATGTTCAAAGAACTCATGGCA |
| Hes5 | ACCGCATCAACAGCAGCATAGAG | ATCTCCAGGATGTCGGCCTTCTC |
| Olig2 | AAGCTCTCCAAGATCGCC | GTAGATCTCGCTCACCAGTC |
| Sox10 | CTGAGCTCAGCAAGACACTAG | GTTGGTACTTGTAGTCCGGATG |
| Id3 | TAACCCAGCCCTCTTCACTTACCC | GCCACCCAAGTTCAGTCCTTCTC |
| Id4 | CGCCCAACAAGAAAGTCAGCAAAG | CAGCAAAGCAGGGTGAGTCTCC |
| Notch1 | AACGAGTGTGAGTCCAACCC | ACGTGGCTCCTGTATATGGC |
| Dlx1 | AGACTTCATCTGACGCTGAGTGTTG | TCCTGTCCTTGTTCCCTCTTCTGG |
| GAPDH | GGCAAATTCAACGGCACAGTCAAG | TCGCTCCTGGAAGATGGTGATGG |
| Gad67 | GCCACAAACTCAGCGGCATAGAAA | AGACGTCATACTGCTTGTCTGGCT |

| **Supplementary table 3 Sequences of primers used for CUT&RUN-qPCR** | | |
| --- | --- | --- |
| **Name** | **Forward (5’-3’)** | **Reversed (5’-3’)** |
| Promoter | ACAGCGTTGACGGAATGGAGTG | TGACCAGCCAATCAGGAGGACAG |
| PDR | CCTGGGATGCTGTTTGAAACTTGC | CCGATCTGGCTGCTGAAGTCAC |
| PDR, promoter-deprived region | | |
