# Supplementary figures and images for: Deubiquitinase Mysm1 regulates neural stem cell proliferation and differentiation by controlling Id4 expression
Source: Cell Death Dis. 2024 Feb 12;15(2):129. doi: 10.1038/s41419-024-06530-y (PMC10859383; doi:10.1038/s41419-024-06530-y)

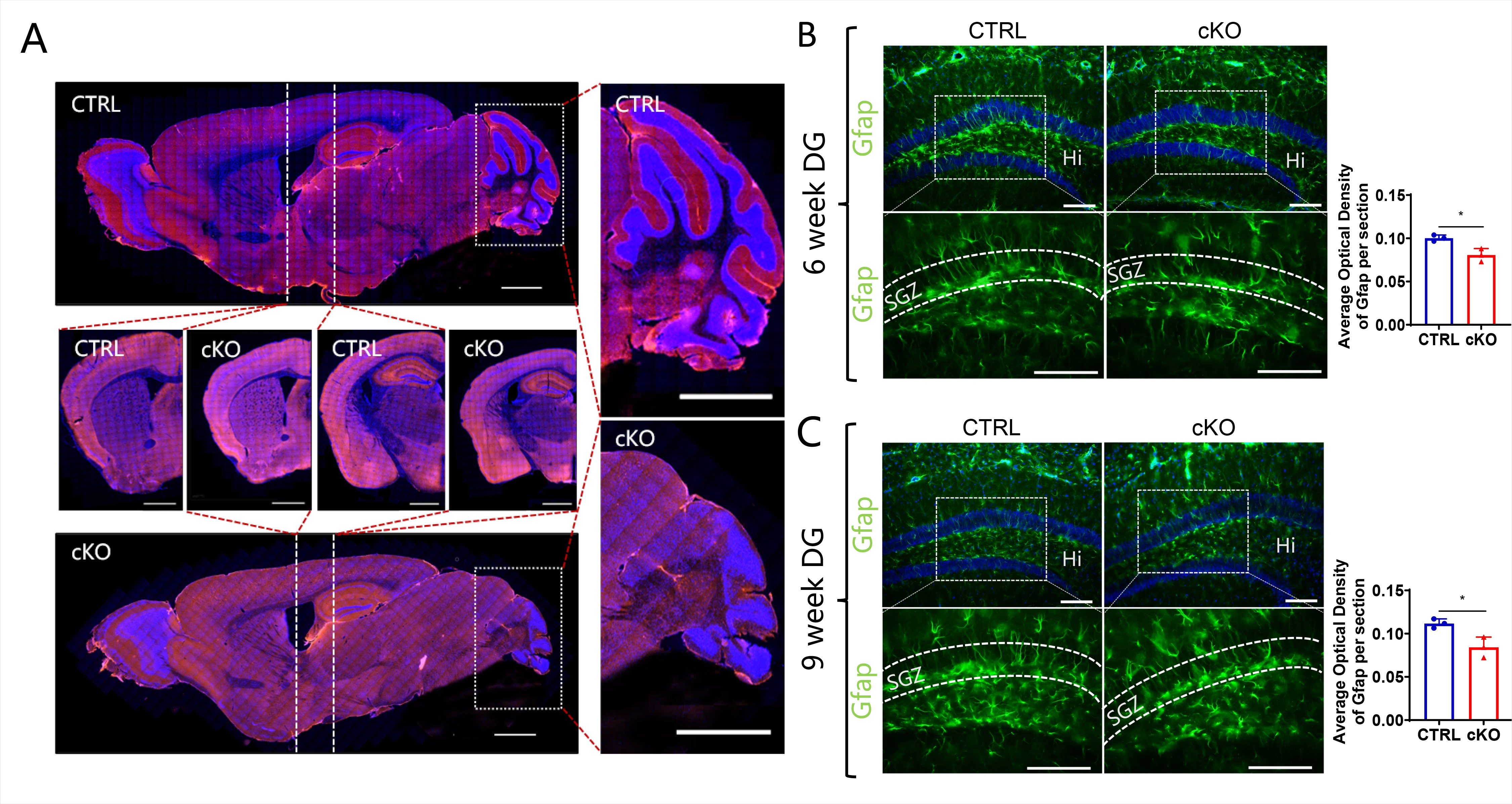

Supplement: Supplementary file 2 — Supplementary Figure 1 [file 41419_2024_6530_MOESM2_ESM.jpg]

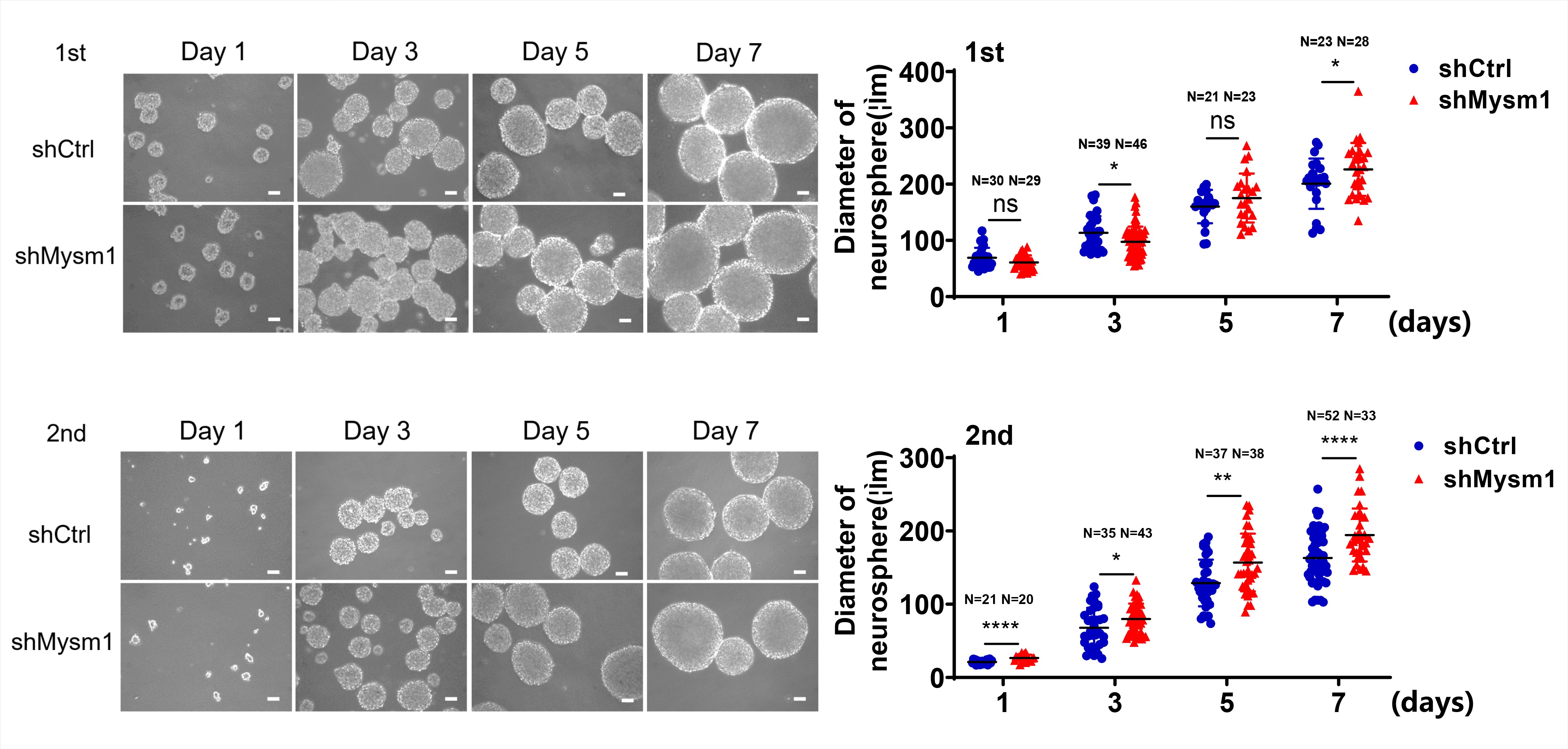

Supplement: Supplementary file 3 — Supplementary Figure 2 [file 41419_2024_6530_MOESM3_ESM.jpg]

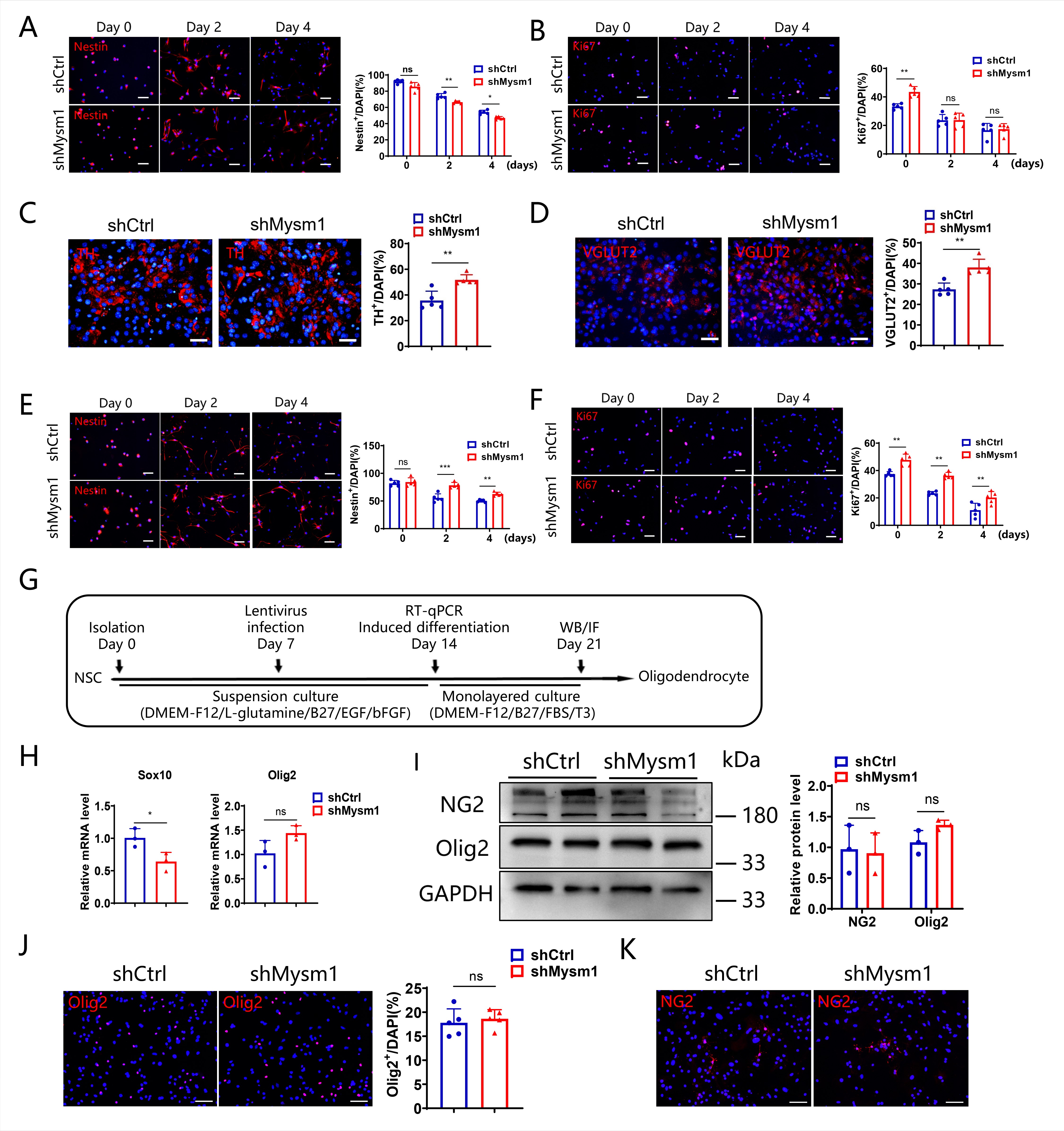

Supplement: Supplementary file 4 — Supplementary Figure 3 [file 41419_2024_6530_MOESM4_ESM.jpg]

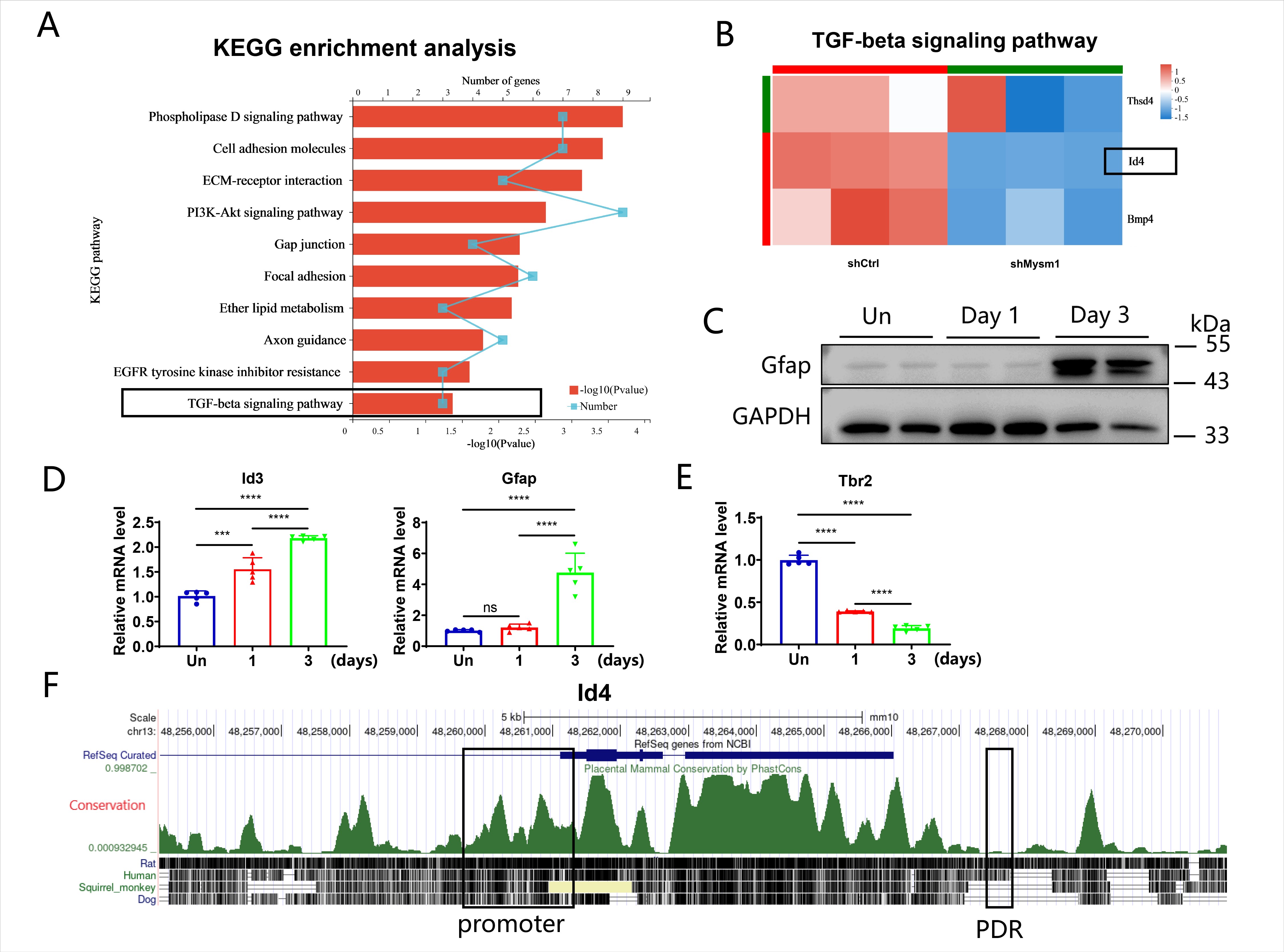

Supplement: Supplementary file 5 — Supplementary Figure 4 [file 41419_2024_6530_MOESM5_ESM.jpg]

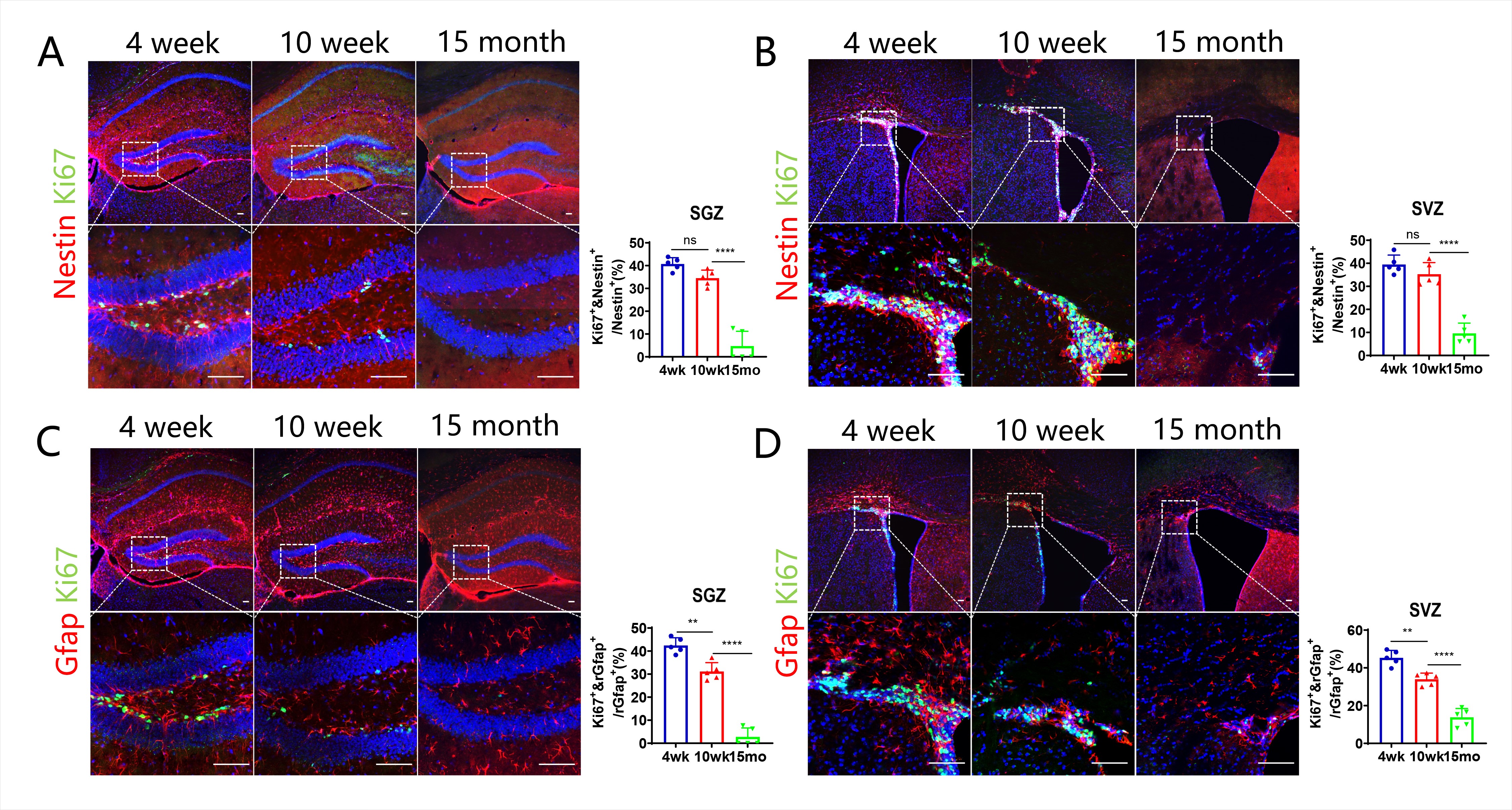

Supplement: Supplementary file 6 — Supplementary Figure 5 [file 41419_2024_6530_MOESM6_ESM.jpg]

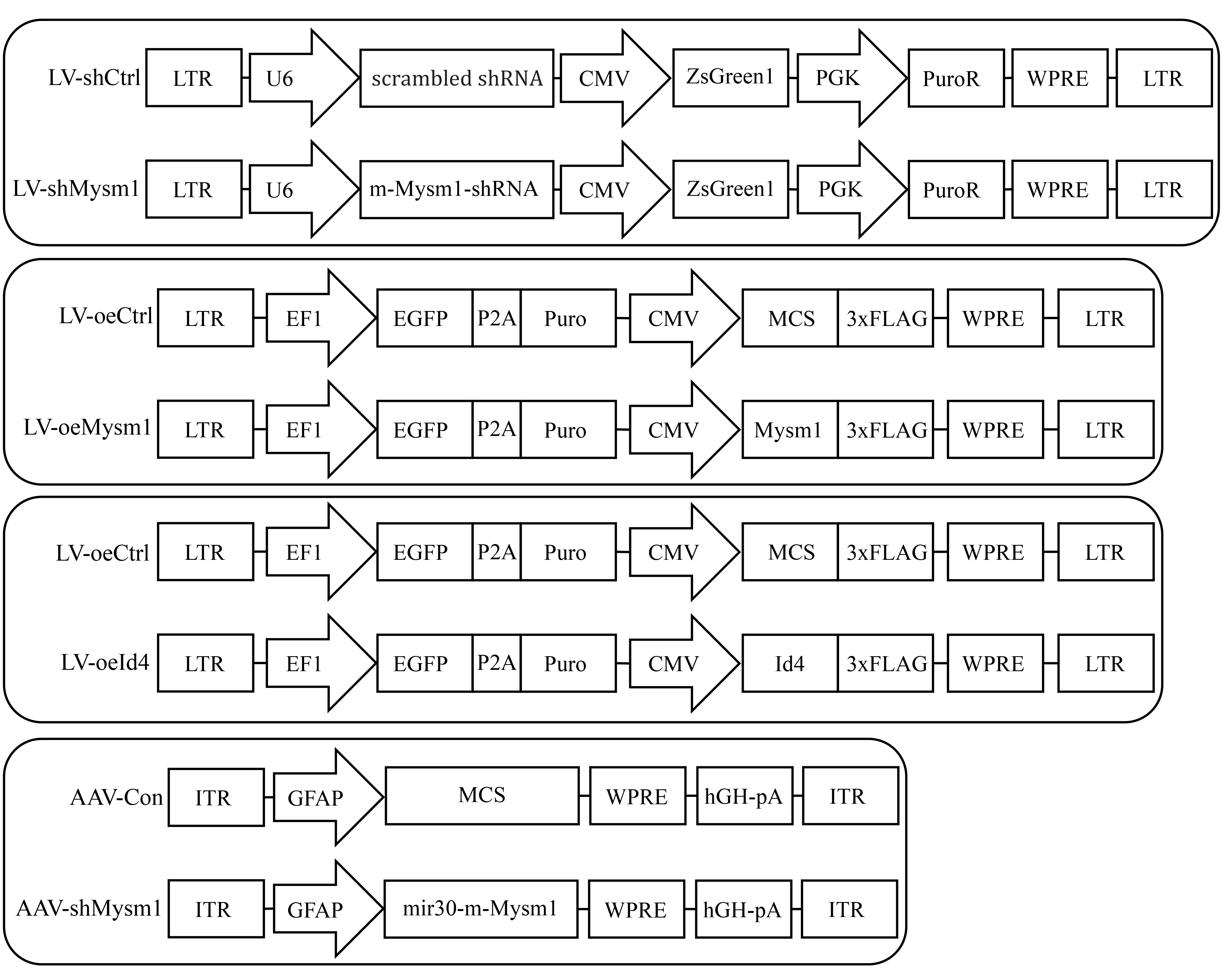

Supplement: Supplementary file 7 — Supplementary Figure 6 [file 41419_2024_6530_MOESM7_ESM.jpg]
