# Supplementary material for: Deubiquitinase Mysm1 regulates neural stem cell proliferation and differentiation by controlling Id4 expression
Source: Cell Death Dis. 2024 Feb 12;15(2):129. doi: 10.1038/s41419-024-06530-y (PMC10859383; doi:10.1038/s41419-024-06530-y)
Supplement: Supplementary file 8 — Original western blots [file 41419_2024_6530_MOESM8_ESM.docx]

**Western Blot**

**Figure 1E**

Mysm1 β-Actin

**
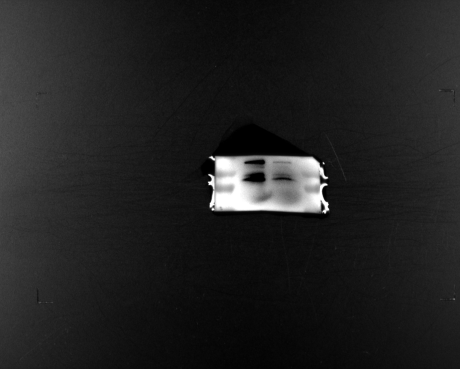

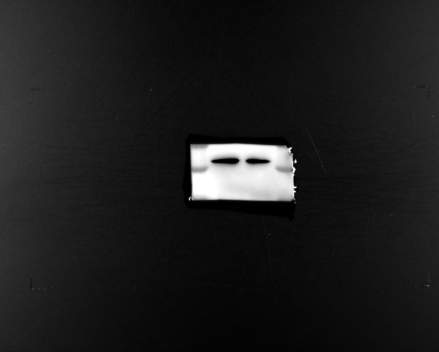
**

**Figure 2A**

GAPDH Mysm1


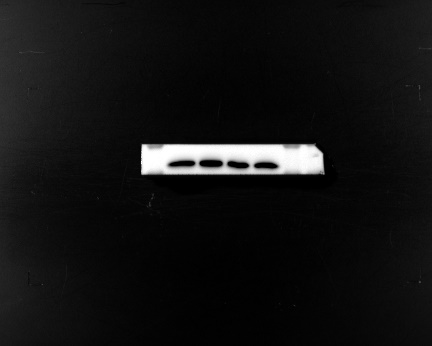

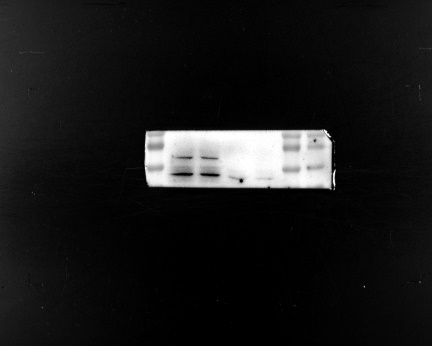


**Figure 2H**

Bax GAPDH

**
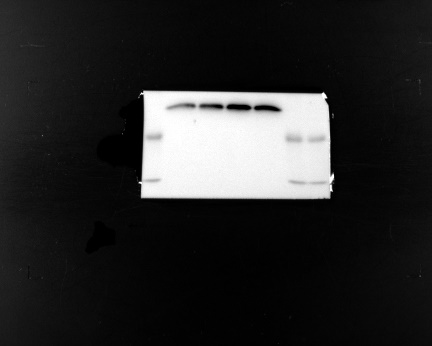

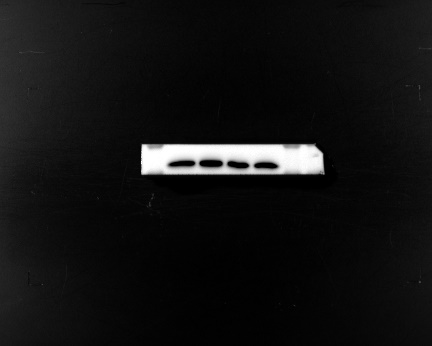
**

Mcm2 p53

**
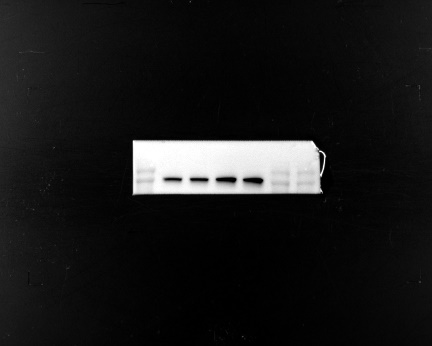

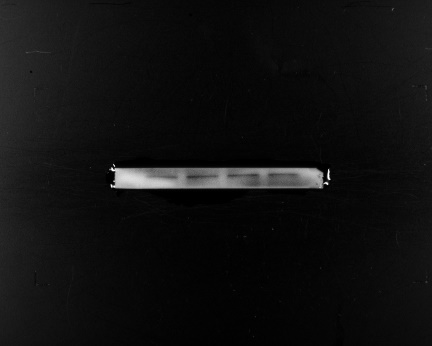
**

p-p53 Puma

**
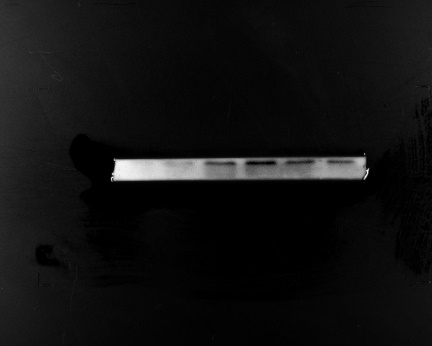

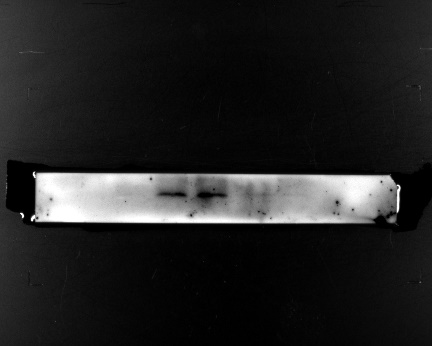
**

**Figure 3C**

GAPDH Dcx

**
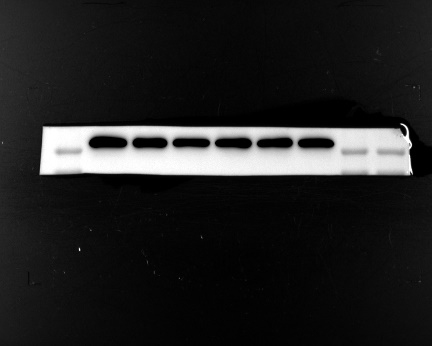

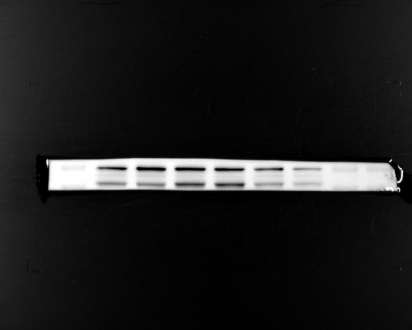
**

Map2

**
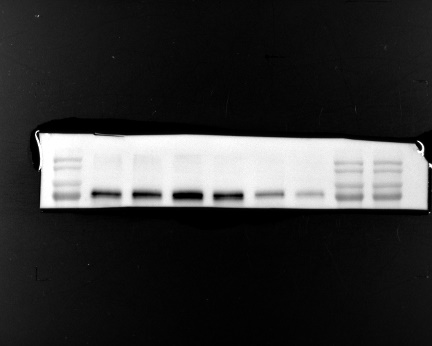
**

**Figure 3J**

GAPDH Aldh1l1

**
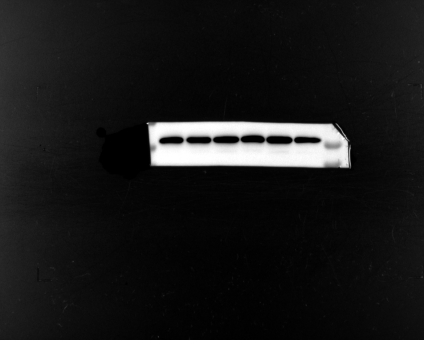

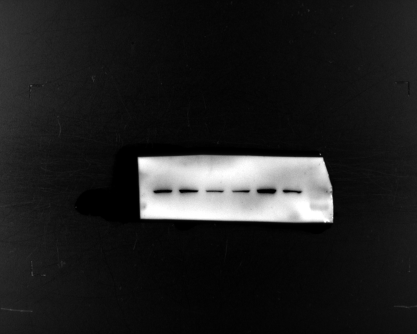
**

Gfap

**
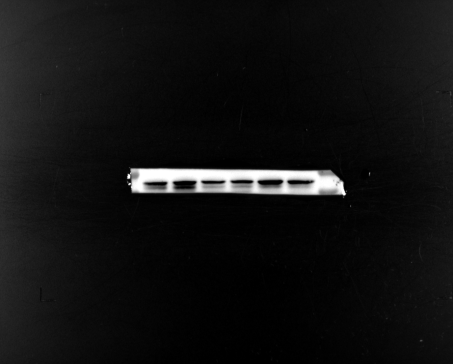
**

**Figure 4E**

GAPDH Gfap

**
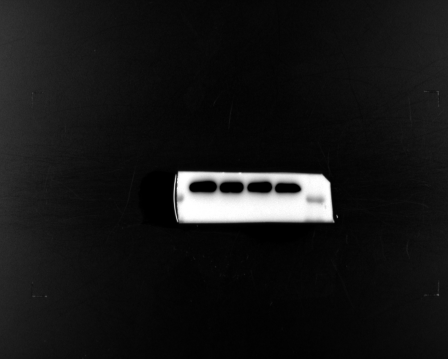

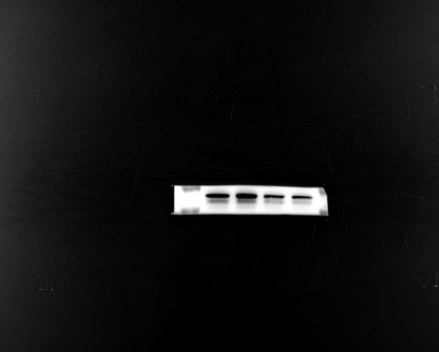
**

Id4


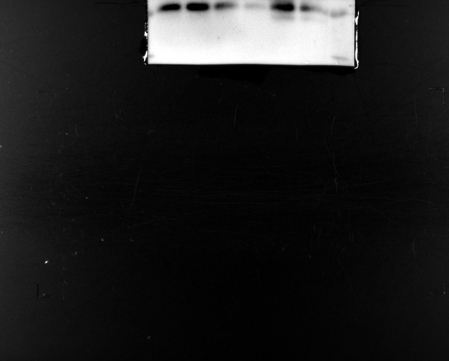


**Figure 4F**

GAPDH Gfap

**
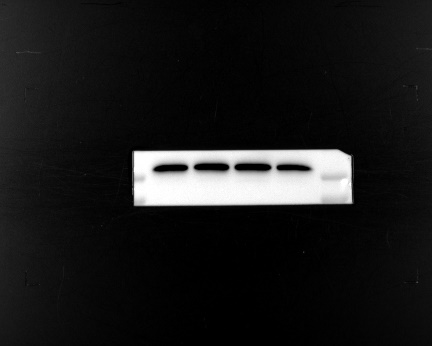

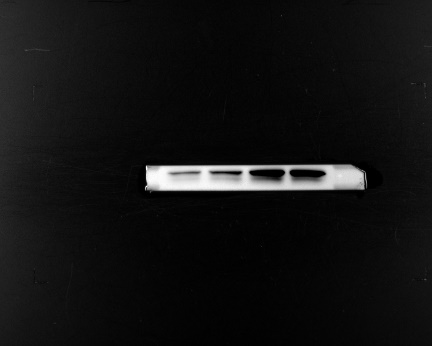
**

Id4

**
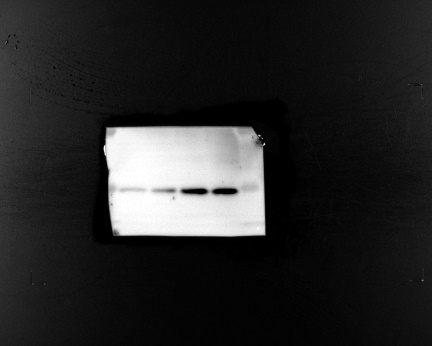
**

**Figure 5A**

GAPDH Id4

**
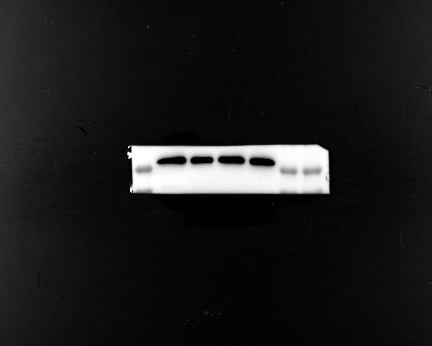

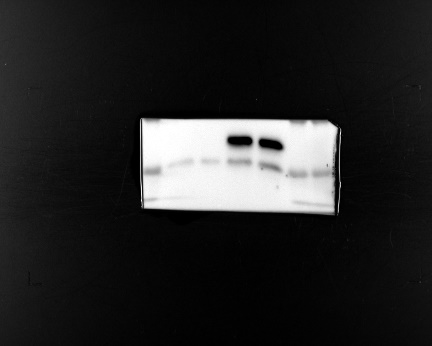
**

**Figure 5D**

GAPDH Bax

**
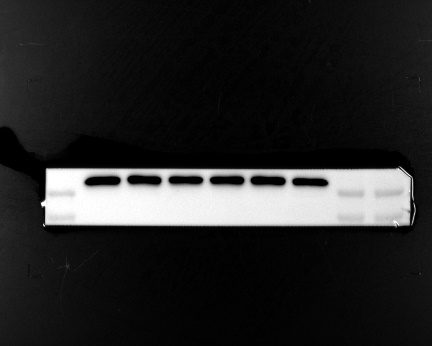

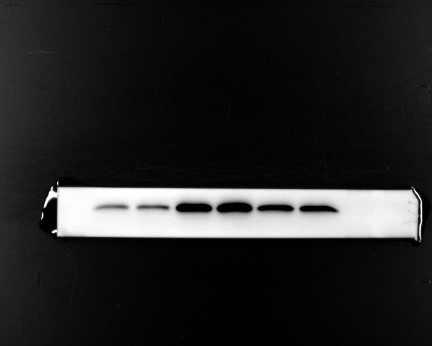
**

**Bcl-2 Mcm2**

**
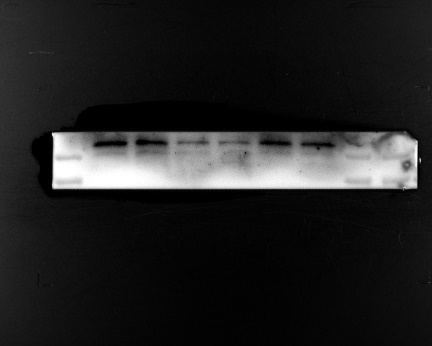

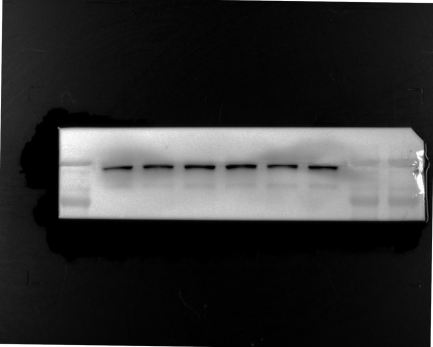
**

**Puma p-p53**

**
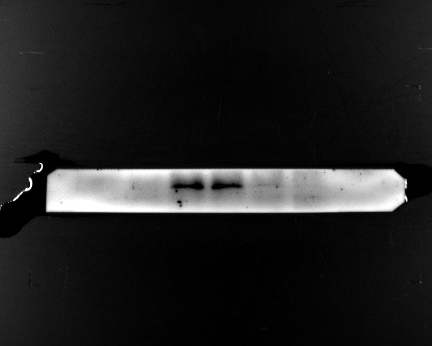

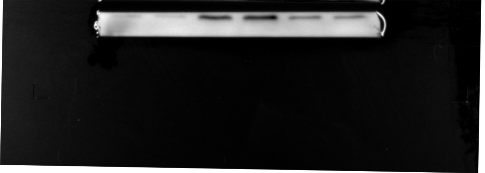
**

**Figure 5I**

GAPDH Dcx

**
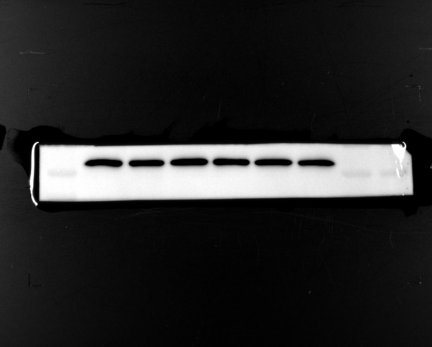

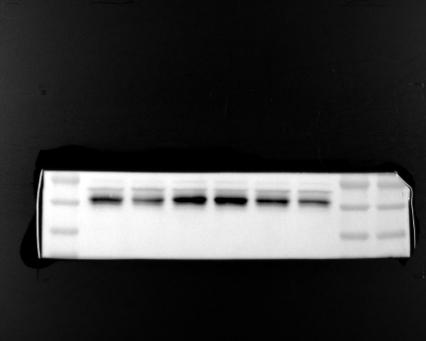
**

Map2

**
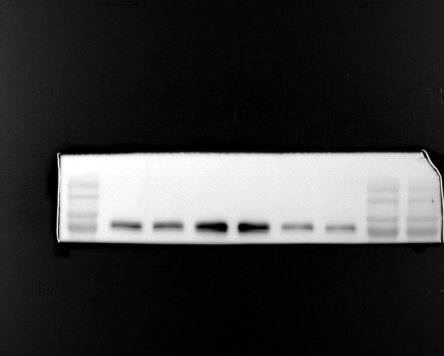
**

**Figure 5N**

GAPDH Aldh1l1

**
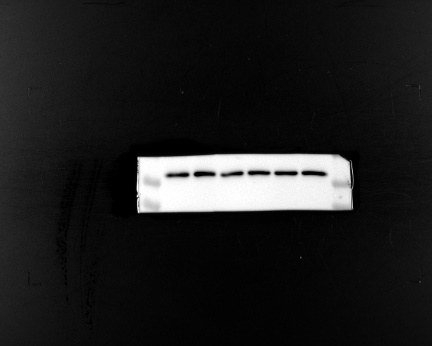

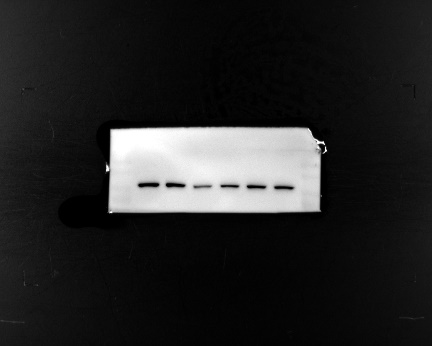
**

Gfap

**
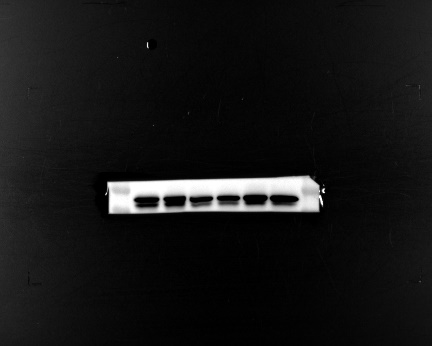
**

**Figure S3 I**

GAPDH NG2

**
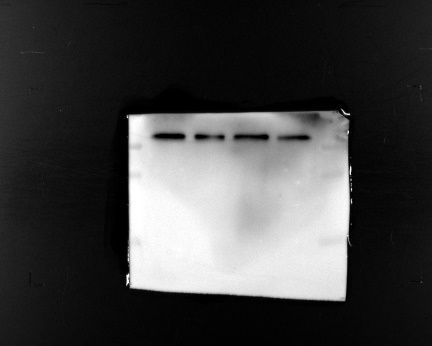

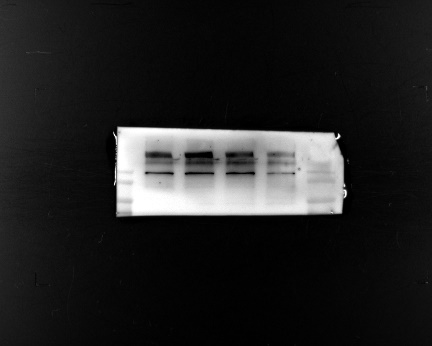
**

Oligo2

**
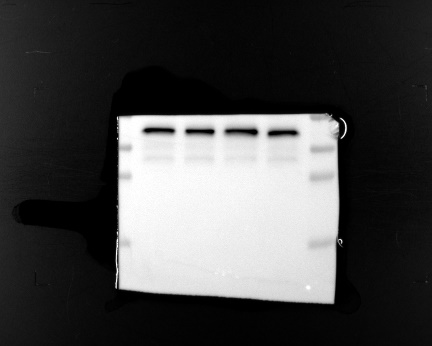
**

**Figure S4 C**

GAPDH Gfap

**
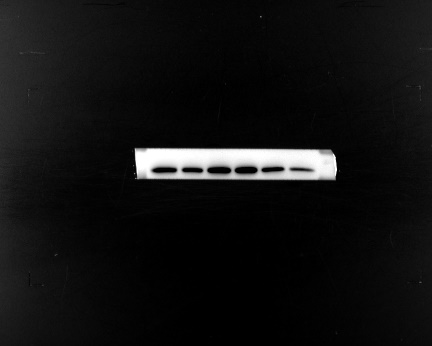

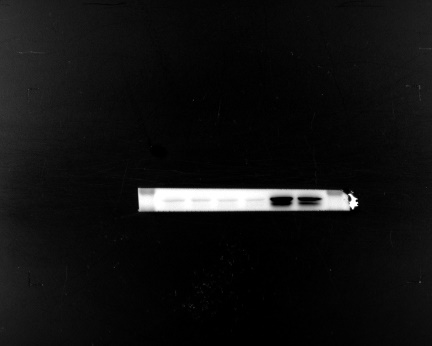
**
